# Electronic hair: Transforming hair into a scalable bioelectronic interface

**DOI:** 10.1126/sciadv.aei3615

**Published:** 2026-11-11

**Authors:** Shweta Shweta, Malamati Giompliaki, Linta Sohail, Rémy Cornuéjols, Preston D. Donaldson, Tobias Abrahamsson, Dina KC, Maria Ntzouni, Sıla Yıldız Cengiz, Sofia Drakopoulou, Mary J. Donahue, Maria Seitanidou, Zifang Zhao, Daniel T. Simon, Magnus Berggren, Sarah Mclntyre, Robrecht Raedt, Xenofon Strakosas, George D. Spyropoulos

**Affiliations:** ^1^ Neural Waves, Department of Information Technology, Ghent University, Technologiepark Zwijnaarde 126, 9052 Zwijnaarde, Belgium.; ^2^ Laboratory of Organic Electronics, Department of Science and Technology, Linköping University, 601 74 Norrköping, Sweden.; ^3^ Core Facility at the Faculty of Medicine and Health Sciences, Linköping University, 601 74 Norrköping, Sweden*.*; ^4^ Center for Social and Affective Neuroscience, Faculty of Medicine and Health Sciences, Linköping University, 601 74 Norrköping, Sweden*.*; ^5^ Department of Neurobiology and Behavior, Cornell University, Ithaca, NY 14853, USA.; ^6^ Wallenberg Initiative Materials Science for Sustainability, Department of Science and Technology, Linköping University, 601 74 Norrköping, Sweden.; ^7^ 4Brain, Department of Head and Skin, Ghent University, 9000 Ghent, Belgium*.*

## Abstract

In electroencephalography, hair remains a major barrier to electrode-skin contact, limiting signal quality. Here, we introduce electronic hair (eHAIR), a strategy that converts hair into a bioelectronic interface using mixed ionic-electronic conductive polymer dyes. Hair, a keratinized filament anchored within the skin, provides a stable and naturally distributed platform for interfacing. Two complementary approaches based on polythiophene derivatives enable rapid, washable functionalization of hair bundles or semipermanent coating of individual strands. The resulting eHAIR forms conformal, low-impedance interfaces that support electrophysiological recordings with increased signal amplitude and spectral power while preserving the underlying neural dynamics without gels or rigid hardware. Beyond passive sensing, eHAIR-based organic electrochemical transistors enable local signal amplification, with gain defined by hair geometry. By repurposing hair as an active bioelectronic interface, eHAIR establishes a wearable and biointegrated platform that can be implemented from individual hairs to larger hair-covered regions.

## INTRODUCTION

Organic bioelectronics use soft, mixed ion-electron conductors to transduce biological signals into electronic readouts, enabling biocompatible, high-resolution neural interfaces (*1*–*3*). These materials have advanced electrophysiology across the full spectrum of invasiveness, from deep brain probes to cortical surface arrays and electroencephalography (EEG) (*4*–*7*), by lowering impedance (*8*, *9*), improving volumetric capacitance (*10*–*12*), and enabling local amplification through organic electrochemical transistors (OECTs) (*13*–*16*). However, most high-performance interfaces still rely on substrates and cleanroom fabrication, creating persistent mechanical mismatches with soft, three-dimensional tissues and limiting scalable, user-friendly neurotechnologies.

Recent efforts have explored substrate-free conducting polymers formed directly within tissue to create hybrid bioelectronic interfaces (*17*). While these approaches improve biointegration, they have so far been largely focused on invasive neural implants, with minimal progress toward noninvasive electrophysiology. In EEG, fundamental challenges persist: Hair obstructs electrode-skin contact, and the high-impedance stratum corneum further degrades signal quality (*18*, *19*). Existing solutions—including conductive gels, abrasive skin preparation, and microfabricated dry electrodes—are often impractical for long-term or real-world use (*18*, *20*–*25*). Together, these challenges highlight the absence of a scalable, conformal, and user-friendly interface for high-quality noninvasive electrophysiology.

Here, we introduce a fundamentally different electrophysiological interface that transforms hair from a barrier into a functional bioelectronic interface (Fig. 1A). Hair consists of a keratinized shaft anchored within the dermis and hypodermis, providing a naturally distributed and mechanically compliant structure. Its surface is composed of keratin cuticles rich in functional groups (─NH_2_, ─COOH, ─OH, and ─SH), offering multiple binding sites for material integration. We leverage this architecture to form electronically active coatings directly on hair using mixed ionic-electronic conductive polymer dyes (eDYEs). Two complementary approaches based on polythiophene derivatives are developed. A top-down method uses a poly(3,4-ethylenedioxythiophene) polystyrene sulfonate (PEDOT:PSS)-polydopamine (PDA) composite to form conformal coatings on individual or bundled hairs (eDYE_C_) through PDA prefunctionalization (Fig. 1B, top, and fig. S1). This approach produces highly conductive, low-impedance coatings that are readily applied and removed, making them well suited for rapid, on-demand electrophysiological recordings. In parallel, a bottom-up strategy uses enzymatically catalyzed polymerization of PEDOT-COONa to grow conductive polymers directly on single hair strands (eDYE_P_) (Fig. 1B, bottom, and fig. S1). Because polymerization occurs locally at the hair surface, each strand is functionalized independently, enabling semipermanent, strand-specific conductive coatings suitable for extended monitoring. Together, these strategies provide complementary routes spanning temporary, washable interfaces to longer-lasting, localized functionalization while preserving the natural architecture of hair. eHAIR can be fully compatible with existing EEG hardware, where functionalized hair can serve as extended dry electrodes and low-resistance interconnects, while conventional metallic electrodes are retained only as electrical contacts to the recording system. Both eDYEs can be directly applied to hair; however, the native hair surface is typically covered by a thin lipid layer that imparts hydrophobicity and reduces affinity for aqueous formulations (*26*, *27*). Prewashing to expose the underlying keratin structure enhances coating adhesion (see Materials and Methods). These approaches yield stable, low-impedance, and highly conductive interfaces that enable gel-free, comfortable EEG acquisition and direct compatibility with conventional hardware and human hair and skin. Hair thus serves simultaneously as an electrode, interconnect, and structural support. Beyond passive sensing, hair-integrated OECTs enable local signal amplification with gain intrinsically defined by the hair geometry. Together, these results establish hair as an active, substrate-free bioelectronic interface and introduce a scalable platform for biointegrated, user-friendly neural monitoring.

**Fig. 1.**
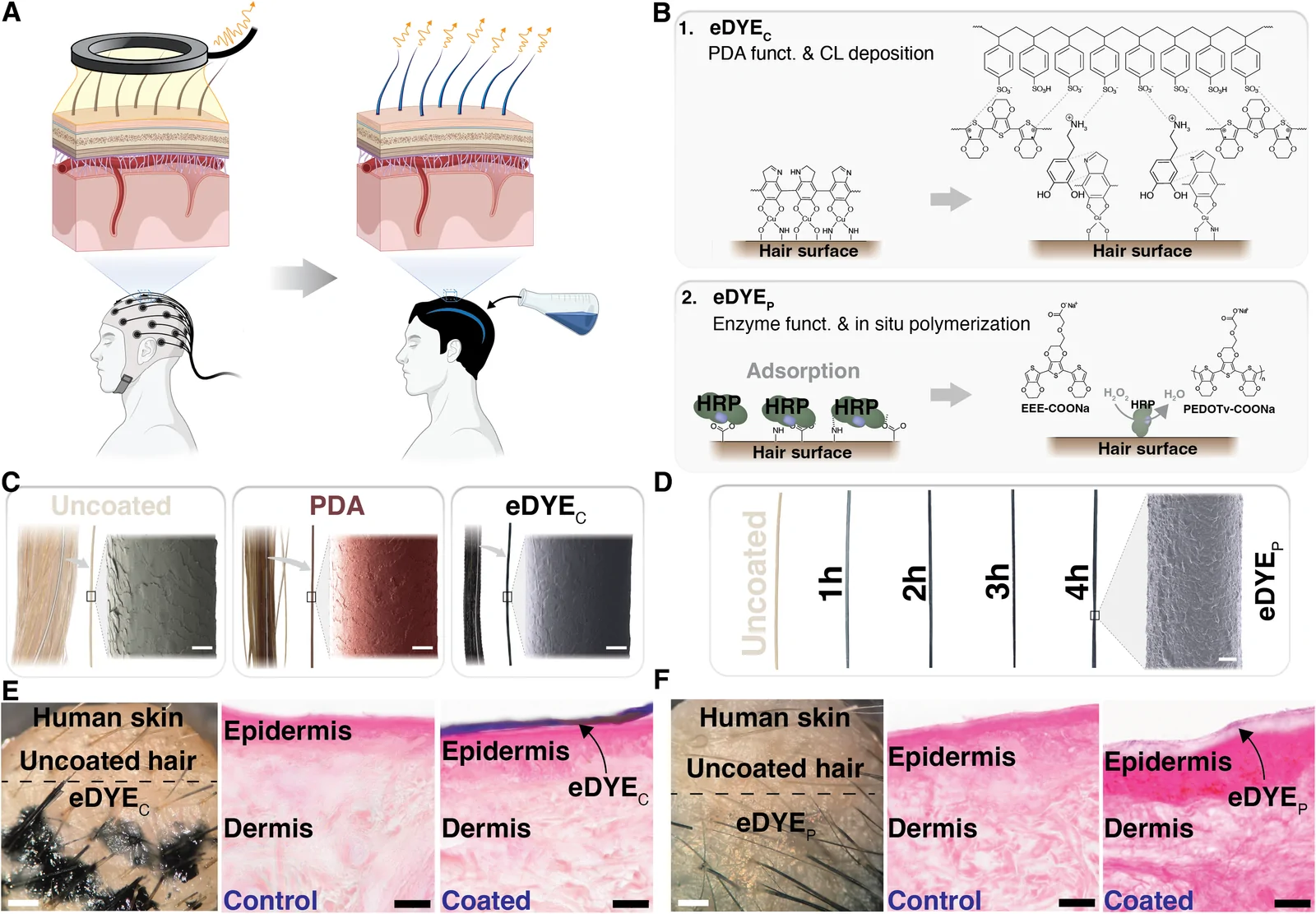
Electronic hair interfaces enabled by two complementary eDYE strategies. (**A**) Schematic illustration comparing conventional EEG electrodes (left) with the eDYE approach (right), in which conductive formulations are directly applied onto the hair to create recording sites without gels, skin abrasion, or rigid hardware. (**B**) Overview of the two eDYE methodologies: (1) eDYE_C_: Hair is functionalized with a PDA adhesion layer, followed by deposition of a PEDOT:PSS:DA composite, forming a conformal conductive coating. (2) eDYE_P_: HRP is immobilized onto the hair surface and subsequently used to catalyze in situ oxidative polymerization of EEE-COONa, resulting in PEDOT-COONa directly within the hair structure. (**C**) Optical micrographs and color-coded SEM images of uncoated, PDA-coated, and eDYE_C_-coated hairs (scale bars, 20 μm). (**D**) Optical images of hair strands after 1 to 4 hours (h) of enzymatic polymerization, showing progressive darkening and formation of a conductive layer. SEM image of the 4-hour sample (scale bar, 20 μm). (**E**) eDYE_C_ interface with human hair and skin. Left: Photograph of eDYE_C_-coated bleached human hair and skin (bottom part). Scale bar, 1 mm. Right: Histological sections (eosin staining) of control and coated skin, showing no visible tissue damage or toxicity and a conformal interface extending from the coated hair toward the epidermis and surrounding the hair root region (scale bars, 50 μm). eDYE_C_ promotes a seamless interface between the electrode and the skin. (**F**) eDYE_P_ interface with human hair and skin. Left: Photograph of eDYE_P_-coated bleached human hair and skin (scale bars, 1 mm). Right: Histological sections (eosin staining) of control and coated skin, showing no visible tissue damage or toxicity. Unlike eDYE_C_, eDYE_P_ forms discrete particles rather than a continuous film on the skin surface. This shows that the coating remains specific to the hair structure. Scale bar, 50 μm. Panel (A) was partially created in BioRender. Spyropoulos, G. (2026) https://BioRender.com/6inggky.

## RESULTS

### Method 1: eDYE_C_—PDA functionalization and conductive layer deposition

In method 1, individual hair strands were first functionalized with a PDA layer to create an adhesive and chemically active interface for subsequent conductive coating. PDA deposition was achieved using two established polymerization routes (see Materials and Methods and fig. S2). In the catalyst-assisted approach, dopamine oxidation was accelerated using CuSO_4_ and H_2_O_2_, generating reactive oxygen species that promote rapid PDA formation (*28*). The presence of Cu^2+^ ions further enhances adhesion through metal coordination, yielding a uniform and mechanically robust coating (of 35 to 45 nm in thickness) within ∼5 min, substantially faster than conventional PDA deposition (*29*–*31*), while maintaining comparable structural characteristics (Fig. 1C). Optical microscopy and scanning electron microscopy (SEM) confirmed continuous, homogeneous coverage of the hair cuticle (Fig. 1C). Quantitative analysis of normalized RGB (red, green, and blue) intensity variation revealed a pronounced contrast change (∼55%) after 5 min, consistent with formation of a dense surface layer (fig. S3). As an alternative, catalyst-free dopamine self-polymerization in alkaline tris buffer (pH ∼8.5) produced similarly uniform PDA coatings via spontaneous oxidation and self-assembly driven by covalent and noncovalent interactions, including hydrogen bonding, π-π stacking, electrostatic interactions, and van der Waals forces (*32*, *33*). Although both approaches yielded uniform PDA layers (fig. S4), the catalyst-assisted protocol provided substantially faster kinetics and improved adhesion efficiency and was therefore used in subsequent experiments, unless otherwise noted.

Following PDA functionalization, the eDYE_C_ interface was completed by depositing a conductive PEDOT:PSS-dopamine hydrochloride (DA-HCl) layer (fig. S5). The interaction between dopamine and PSS chains strengthens ionic pairing, facilitating ammonium (─NH_3_^+^) and sulfonate (─SO_3_^−^) interactions. This process promotes more ordered stacking of PEDOT chains and improves phase separation between PEDOT and PSS, enhancing intermolecular packing through DA-PSS interactions and ultimately improving charge transport (*34*). As a result, the PEDOT:PSS:DA films exhibit substantially higher conductivity compared to pristine PEDOT:PSS (*34*). The conductive formulation was applied to PDA-coated hair strands using a brush, followed by mild thermal treatment (30° to 90°C) using a hair dryer to accelerate solvent evaporation and promote polymer annealing (fig. S6). The resulting coatings (∼8 μm in thickness after five coating cycles) were uniform, flexible, and mechanically compliant. Where a high spatial resolution was required, an optional insulating layer was introduced to electrically isolate adjacent hairs, as discussed later in the manuscript. Electrical interfacing was achieved either through direct mechanical contact using metallic clips or via seamless integration with flexible cables through overlapping eDYE layers, both yielding stable signal transmission with minimal impedance variation (fig. S7).

### Method 2: eDYE_P_—Enzyme functionalization and in situ polymerization

In method 2, conductive coatings were formed on individual hairs through a stepwise enzymatic polymerization process that grows a mixed ionic-electronic conductor directly on the hair surface (Fig. 1B and fig. S1, bottom). Hair strands were first functionalized with oxidoreductase enzymes, including horseradish peroxidase (HRP) or bilirubin oxidase (BOx), or enzyme cascades, such as glucose oxidase/HRP (GOx/HRP) or lactate oxidase/HRP (LOx/HRP), via physical adsorption during a dip-coating step. Each enzyme system uses a distinct polymerization input: HRP uses H_2_O_2_, BOx relies on molecular oxygen, and GOx/HRP or LOx/HRP systems generate H_2_O_2_ in situ from endogenous metabolites (glucose or lactate, respectively) (fig. S8). To further confine polymer growth to individual hairs, enzymes were optionally covalently immobilized within the keratin matrix using EDC/sulfo-NHS [1-ethyl-3-(3-dimethylaminopropyl) carbodiimide/*N*-hydroxysulfosuccinimide] coupling chemistry. Following enzyme functionalization and washing, a solution containing the water-soluble monomer precursor sodium 2-((2,2′,2″,3,3′,3″-hexahydro-[5,5′:7′,5″-terthieno[3,4-*b*][1,4]dioxin]-2′-yl)methoxy)acetate (EEE-COONa) and H_2_O_2_ was applied to the hair. Enzymatic oxidation generates monomer radicals that polymerize further in situ, resulting in the growth of a PEDOT-COONa organic mixed ionic-electronic conductor directly on the hair surface.

Optical microscopy and SEM imaging of blonde hair revealed uniform, high-surface-area polymer coatings (fig. S9) accompanied by a progressive color change indicative of localized polymer growth, which saturated after ∼3 hours (Fig. 1D). Quantitative RGB intensity analysis showed rapid initial growth, increasing from ∼20% after 1 hour to ∼55 to 60% after 2 hours, followed by a plateau (∼65%) at longer polymerization times, consistent with localized enzymatic growth, which saturates after 3 hours (fig. S3, C and D). After rinsing, the hair retained a darker appearance, confirming stable polymer deposition. Postpolymerization EDC/sulfo-NHS treatment was optionally applied to further enhance coating stability through covalent attachment to the underlying hair structure or immobilized enzymes. This strategy is not limited to a specific hair type or monomer chemistry. Screening of trimeric thiophene-based electrolyte monomer analogs identified EEE-COONa as yielding the highest conductivity; it was therefore selected for subsequent studies (fig. S10) (*17*, *35*, *36*).

Both eDYE formulations are compatible with human hair and exhibit good biocompatibility with human skin. In human skin samples containing hair, eDYE_C_ forms conformal coatings on individual and bundled hairs and extends toward the epidermal surface, creating a homogeneous electrode that establishes a seamless, low-impedance interface without penetrating or altering the underlying tissue (Fig. 1E). Histological evaluation (experimental details are provided in Materials and Methods) using eosin staining revealed no visible signs of tissue damage, irritation, or inflammation. Hematoxylin staining was not performed because its blue-purple contrast would obscure the dark eDYE coatings. These observations are consistent with the established biocompatibility of both PDA and PEDOT:PSS, which have been extensively investigated as biointerface materials in previous studies (*30*, *37*, *38*). In contrast, eDYE_P_ produces coatings that remain confined to the hair structure, extending along the shaft and potentially reaching the hair follicle and root, with no detectable continuous film on the skin surface (Fig. 1F). Similarly, histological analysis showed no visible signs of tissue disruption or toxicity, in agreement with previous studies demonstrating the biocompatibility of EEE-COONa and its polymerized derivatives (*39*, *40*).

### Process optimization and electrical and electrochemical characterization

We first established conductive coatings on hair and characterized their electrical and electrochemical properties. To validate the efficacy of conductive eDYEs for electrophysiological signal extraction, we performed a comprehensive set of electrical and electrochemical characterizations, including electrochemical impedance spectroscopy (EIS), cyclic voltammetry (CV), current-voltage (*I*-*V*) measurements in dry conditions, and four-point probe measurements.

#### Method 1 (eDYE_C_)

Repeated coating cycles of PEDOT:PSS:DA layer on PDA-functionalized hairs resulted in a systematic reduction of electrical resistance, both for single hair strands and bundles (Fig. 2A). Resistance decreased monotonically with successive coating cycles, reaching subkilohm values after three iterations, consistent with progressive film thickening and improved percolation of the conductive network. Beyond four coating cycles, the decrease became marginal, suggesting that the measured resistance approached a regime dominated by contact resistance rather than the intrinsic resistance of the conductive coating, as commonly observed for highly conductive PEDOT:PSS films. This interpretation is further supported by EIS, which revealed a similar progressive reduction in impedance with increasing coating cycles before reaching a plateau after approximately four to five coatings (fig. S11), indicating that additional material deposition provides only limited improvements in electrical performance. This resistance trend was consistent across one-, two-, and three-hair configurations, confirming reproducible coating buildup. Dry-state *I*-*V* characteristics exhibited linear, ohmic behavior over the measured voltage range (Fig. 2B). Increasing the number of coated hairs resulted in a proportional increase in current, consistent with parallel conductive pathways. The corresponding electrical resistance, extracted directly from the slope of the linear *I*-*V* curves and plotted as a function of hair number (Fig. 2C), decreased monotonically with increasing number of hairs, approaching a plateau at higher bundle sizes. This behavior indicates that each coated hair acts as an independent conductive microwire, while bundles operate as parallel resistive networks. Minor deviations from ideal inverse scaling likely arise from finite contact resistance, hair-to-hair variations in coating thickness, and/or nonuniform current distribution within the bundle.

**Fig. 2.**
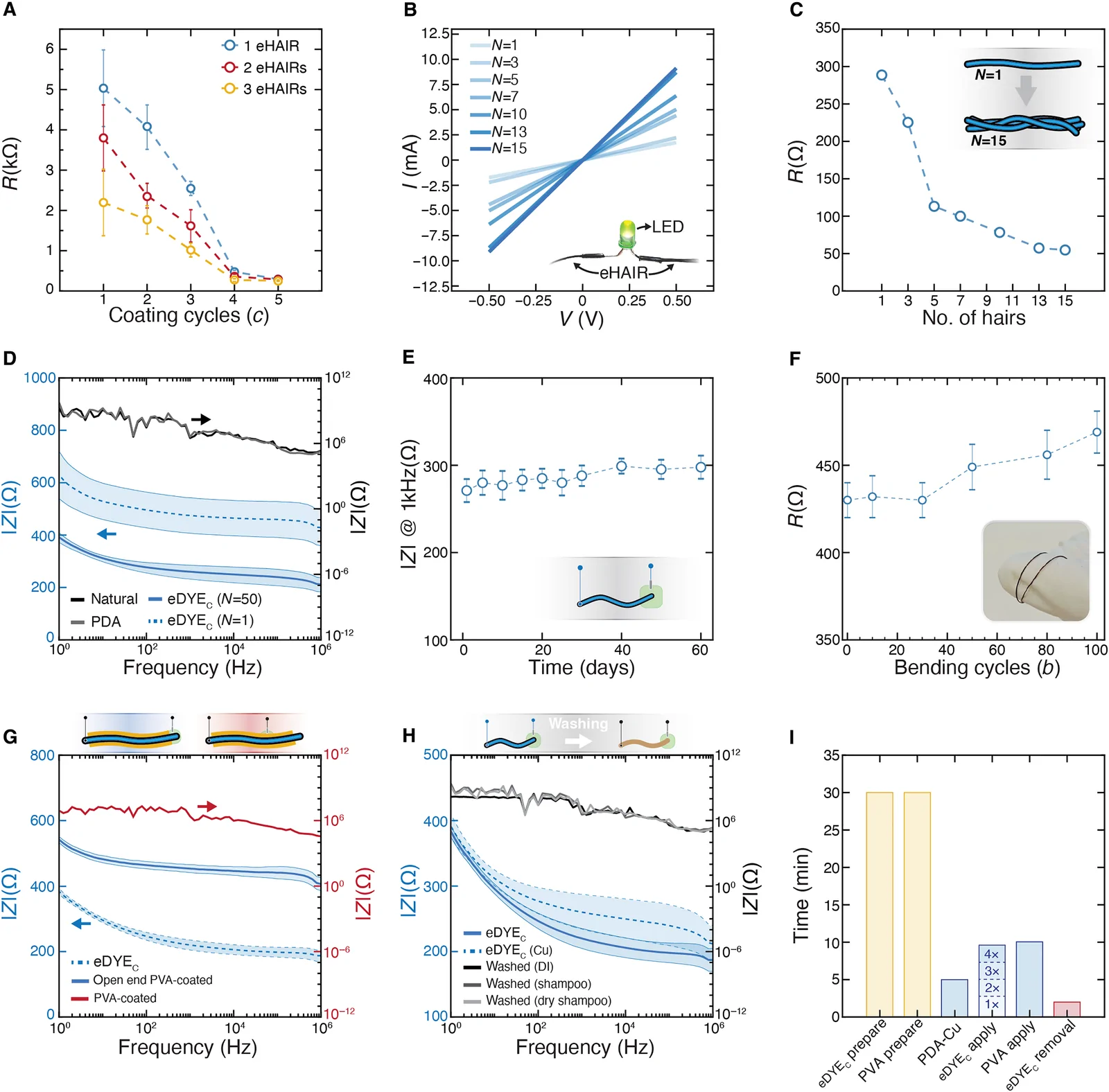
Electrical and mechanical performance of eDYE_C_-coated hair electrodes. (**A**) Electrical resistance as a function of the number of sequential eDYE_C_ coating cycles on the PDA-functionalized hair measured for one (blue), two (red), and three (orange) eHAIRs (*n* = 3). (**B**) *I*-*V* characteristics of eDYE_C_-coated hair bundles with varying numbers of individual hairs (*N* = 1 to 15) (*c* = 5 coating cycles). The inset demonstrates green LED illumination driven at 6 V using eDYE_C_-coated eHAIRs. (**C**) Resistance extracted from the *I*-*V* curves in (B) as a function of the number of parallel eDYE_C_-coated hairs (*c* = 5). (**D**) EIS comparing natural hair (black line), PDA-coated hair (gray line), and eDYE_C_-coated (*c* = 5) single hair strands (dashed blue line; electrode area: 0.4 mm^2^) and bundles of ∼50 hairs (solid blue line; area of electrode: 3 mm^2^). The solid/dashed line indicates the mean; the shaded area represents ±SD (*n* = 5). (**E**) Long-term ambient stability of eDYE_C_-coated (*c* = 5) eHAIRs (∼50 hairs) (*n* = 5). (**F**) Resistance of eDYE_C_-coated (*c* = 5) hair under repeated bending cycles. The inset shows eHAIR bent around a finger (*n* = 5). (**G**) Effect of PVA insulation on eHAIR electrodes. EIS of eDYE_C_-coated hairs (∼50 hairs; *c* = 5) with and without PVA/GOPS coating shows preserved electrode-electrolyte impedance while suppressing lateral electrical cross-talk between adjacent hairs. The solid/dashed line indicates the mean; the shaded area represents ±SD (*n* = 5). (**H**) Washability of eDYE_C_ coatings (*c* = 5). EIS spectra before and after washing with deionized water, shampoo, or dry shampoo indicate that the conductive layer can be removed after detergent washing. The solid/dashed line indicates the mean; the shaded area represents ±SD (*n* = 5). (**I**) Total processing time for each step involved in fabricating eDYE_C_-coated eHAIR.

EIS was performed on single hair strands and hair bundles of 50 hairs (Fig. 2D) over a frequency range of 1 Hz to 1 MHz. Native hair is electrically insulating because of the absence of mobile charge carriers in keratin (*26*, *41*, *42*). Similarly, PDA exhibits low electrical conductivity because of its amorphous nature and the absence of an extended π-conjugated network (*32*, *43*, *44*). Accordingly, both untreated and PDA-treated hairs displayed high impedance values (|*Z*| ∼ 10^5^ to 10^8^ Ω) across the measured frequency range (Fig. 2D). In contrast, eDYE_C_-coated hairs (PDA functionalization followed by five coating cycles of PEDOT:PSS:DA) exhibited a substantial reduction in impedance, reaching ∼500 Ω for single coated hairs and ∼300 Ω for bundles at 1 kHz. These values approach the electrolyte-limited resistance, indicating the formation of a highly conductive (σ = 600 to 1000 S·cm^−1^) (*38*, *45*), high-capacitance (C* = 39 F·cm^−3^) (*46*) interface operating predominantly in a resistive regime at low frequencies (Fig. 2, D and E). Although PDA deposition can be further accelerated under catalytic conditions, this modification had little effect on impedance, confirming that electrical performance is dominated by the PEDOT:PSS:DA layer rather than the underlying PDA film. The capacitive nature of the interface was further supported by CV measurements on single coated hairs, which exhibited high currents in the milliampere range (fig. S12). eDYE_C_-coated hairs exhibited capacitive, box-shaped CV profiles with a capacitance of ∼1.5 mF and no observable redox peaks, indicating that the conductive coating operates predominantly through capacitive charge storage mechanisms in physiological environments (*11*). Such behavior is advantageous for bioelectronic interfaces, as it enables efficient charge transfer while minimizing faradaic reactions and potential tissue damage.

To assess long-term stability, eDYE_C_-coated hairs were monitored for 60 days, during which coated hair strands were kept in a glass petri dish at room temperature under ambient conditions. Impedance remained stable, with <9.88% variation at 1 kHz (Fig. 2E), demonstrating excellent electrochemical and structural stability over time. Mechanical robustness was evaluated through repeated bending, with resistance varying by <10% over 100 bending cycles (Fig. 2F), confirming strong adhesion and mechanical compliance.

Achieving high-spatial-resolution EEG requires that signal localization be governed by physiological volume conduction rather than electrical cross-talk between adjacent interfaces. In a multichannel recording setup, where every channel is composed of eHAIR strands or bundles, neighboring conductive strands could act as unintended lateral conduction pathways, leading to signal mixing and reduced spatial precision. This challenge is particularly critical in high-density EEG, brain-computer interfaces, and functional neuroimaging applications, where precise source localization and channel independence are essential (*47*–*49*). To suppress such interference, we implemented a thin dielectric insulation layer as a proof of concept using poly(vinyl alcohol) (PVA) cross-linked with (3-glycidyloxypropyl)trimethoxysilane (GOPS) (Fig. 2G) (*50*). This strategy demonstrates that nonconductive coatings, potentially including commercially available hair products such as gels or sprays, can be used to electrically isolate adjacent conductive hairs. PVA insulation effectively eliminated lateral electrical coupling between neighboring eHAIRs without degrading the intrinsic electrical performance of the eDYE_C_ coating. Electrochemical impedance measurements showed that insulated eHAIRs retained impedance values comparable to uninsulated eHAIRs (Fig. 2G), demonstrating that lateral cross-talk can be selectively suppressed while preserving the electrode-electrolyte interface.

Beyond electrochemical and electrical performance, practical usability requires that eDYE_C_ remains easy to remove and apply. Similar to commercial temporary hair dyes, the eDYE_C_ coating can be removed within minutes using shampoo. We evaluated two removal approaches, wet shampooing and dry shampoo–based mechanical removal, both of which effectively eliminated the conductive layer. Optical microscopy and EIS confirmed complete loss of conductivity and a return to the native impedance profile of untreated hair (Fig. 2H). While surfactants in shampoo facilitated slightly faster removal, gentle washing with water or dry shampoo alone also proved effective. The underlying PDA adhesion layer remained intact after repeated washing (≥30 cycles) despite complete removal of the conductive PEDOT:PSS:DA coating. PDA itself is electrically insulating but biocompatible and exhibits antibacterial behavior (*30*). The postremoval state thus leaves the user with nonconductive and lightly pigmented (somewhat darkened, but not black; fig. S13) hair. This property helps maintain scalp comfort and biocompatibility while enabling repeated temporary application of eDYE_C_ for short-term or on-demand EEG monitoring. The complete workflow for application and removal of eDYE_C_ can be performed in <30 min, with most of the time associated with solution preparation (∼30 min for eDYE_C_ and PVA formulations; Fig. 2I).

Collectively, these results demonstrate that eDYEC converts hair into a mechanically robust and electrochemically stable mixed-conducting interface (eHAIR), capable of sustained low-impedance operation over extended periods, repeated mechanical deformation, and multiple wash-reapply cycles. The ability to electrically isolate individual strands further enables high-density architectures for improved spatial resolution in EEG. Together, these properties establish eDYE_C_-coated hair as a practical, durable, and reconfigurable platform for long-term or on-demand electrophysiological monitoring.

#### Method 2 (eDYE_P_)

In method 2, coating formation and electrical properties are governed by in situ polymerization kinetics. We therefore systematically investigated the parameters controlling these kinetics and their impact on electrical performance. Unless otherwise stated, polymerization was performed using HRP and H_2_O_2_. We first varied the HRP concentration while fixing the EEE-COONa monomer (2 mM), H_2_O_2_ (1 mM), and polymerization time (3 hours). Higher monomer concentrations provided little improvement in electrical performance, and the selected monomer-to-oxidant ratio was consistent with a prior report (*51*). Increasing HRP concentration led to a pronounced reduction in both electrical resistance and spatial variability along the hair shaft, measured at 5-mm intervals (Fig. 3A). These results indicate the formation of thicker, more uniform PEDOT-COONa coatings. Beyond an HRP concentration of 214 U/ml, resistance values began to plateau (Fig. 3B, black curve), suggesting that polymer growth becomes limited by enzyme accessibility rather than intrinsic enzyme activity, as the available enzyme sites become progressively encapsulated by the growing polymer layer. This interpretation is supported by experiments at higher enzyme loading (535 U/ml), which achieved comparable conductance over shorter polymerization times (Fig. 3B), providing a route to accelerated processing. Furthermore, conductance could be increased by repeating the polymerization cycle on precoated hairs, leading to progressively lower resistance with successive coatings (Fig. 3C), thereby enabling tunable control of hair conductance to meet application-specific requirements.

**Fig. 3.**
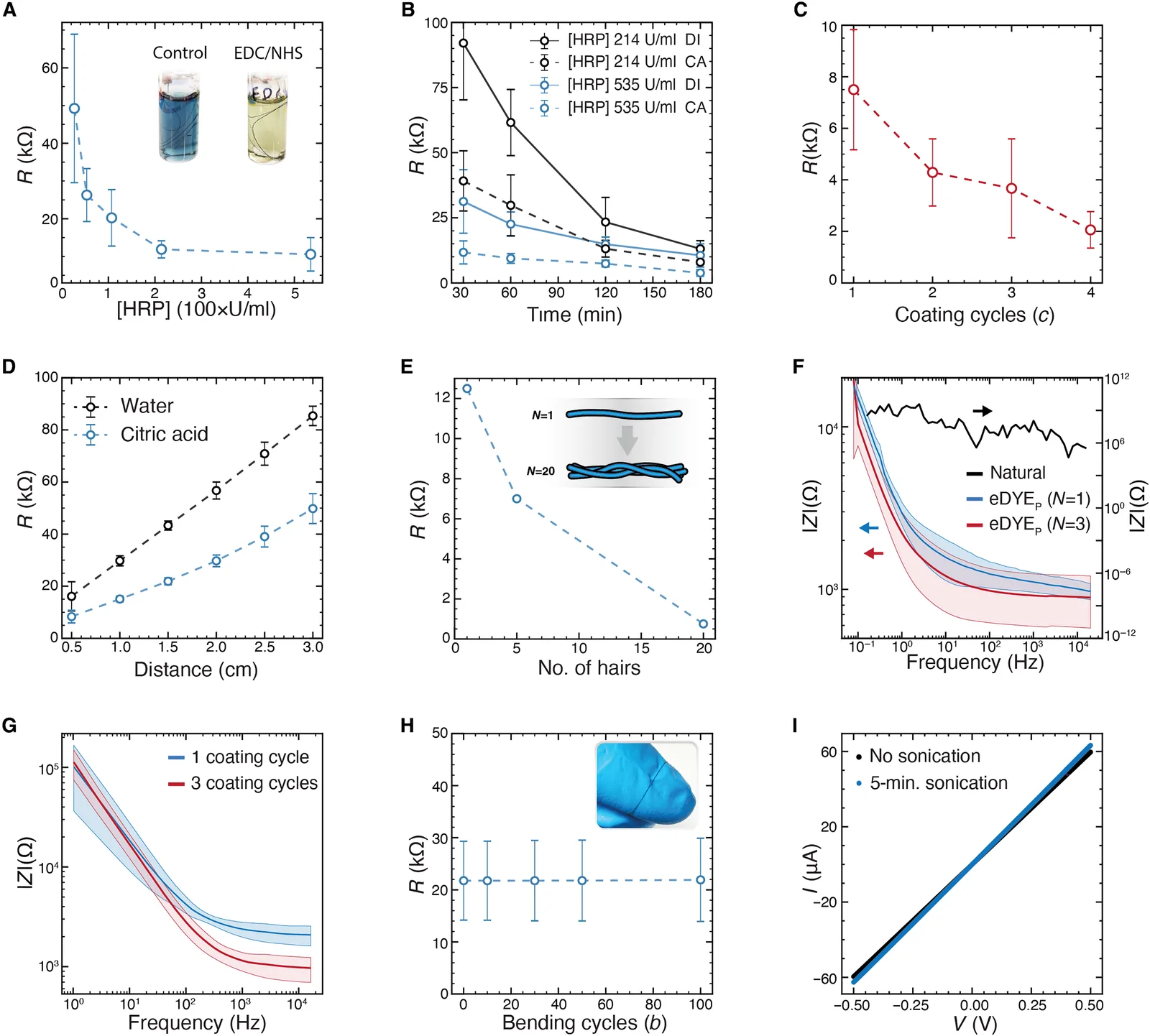
Electrical, mechanical, and chemical performance of semipermanent eDYE_P_-coated eHAIR. (**A**) Effect of HRP concentration on hair resistance following polymerization. The inset shows hair pretreated with HRP immersed in monomer and H_2_O_2_ solution. Control samples lacking EDC/NHS exhibit HRP leaching and polymerization in solution (mean ± SD, *n* = 5 hairs, *m* = 4 measurement segments per hair, *c* = 1 coating cycle). (**B**) Time-dependent evolution of resistance during enzymatic polymerization for two HRP concentrations (214 and 535 U/ml) (solid lines) before and after citric acid (CA) treatment (dashed lines). DI in the legend refers to the di-water washing step (*n* = 5, *m* = 4, *c* = 1). (**C**) Resistance as a function of sequential eDYE_P_ coating cycles using HRP (535 U/ml) after two CA treatments (*n* = 4, *m* = 5). (**D**) Resistance as a function of contact distance along the hair for water-treated and CA-treated samples (*n* = 4, *c* = 1). (**E**) Resistance as a function of the number of eDYE_P_-coated eHAIRs connected in parallel. (**F**) EIS comparing untreated hair with eDYE_P_-coated hair bundles (*N* = 1 and *N* = 3, *c* = 1). The solid line indicates the mean; the shaded area represents ±SD (*n* = 3). (**G**) EIS of eDYE_P_-coated hair as a function of the number of sequential coating cycles using HRP (535 U/ml^−1^) and EEE-COONa (2 mM). Electrode area ≈ 4 mm^2^. The solid line indicates the mean; the shaded area represents ±SD (*n* = 3). (**H**) Resistance after repeated bending cycles of eDYE_P_-coated hair. The inset shows bent hair around a finger (*n* = 3, *c* = 1). (**I**) *I*-*V* characteristics before and after 5 min of detergent sonication. Water rinsing and CA treatment were performed after sonication (*c* = 1).

Conductivity was further enhanced through a posttreatment with citric acid, a mild and biocompatible acid commonly used in cosmetic formulations (*26*). Following polymer deposition, 300 mM citric acid in deionized water (pH 1.5 to 1.8) was applied and dried using a hair dryer on the hot-air setting (60° to 93°C). This treatment was inspired by previous reports showing that both strong and weak acids can enhance the conductivity of PEDOT-based materials (*52*, *53*). Citric acid posttreatment reduced both resistance and its variability (Fig. 3B, dashed curves), with the lowest values observed at higher HRP concentrations. Repeated treatments further decreased resistance over up to three cycles, after which values stabilized (figs. S14 and S15). The resulting electronic hair exhibited uniform coatings along the hair shaft, as evidenced by a linear increase in resistance with hair length (Fig. 3D, black line), while the resistance per unit length decreased following citric acid treatment (Fig. 3D, blue line). This effect is also optically evident from the polymer’s electrochromic response to changes in oxidation state under voltage biasing (movie S1).

Because homogeneous in situ polymerization typically requires 30 to 180 min, we developed a cosmetically inspired gel-based formulation to enable localized application and improve usability. Gels containing either the enzyme or the monomer/H_2_O_2_ were prepared using d-sorbitol (60%, w/w) as a humectant. A first gel containing HRP (or alternative enzymes), EDC/sulfo-NHS, and sorbitol was applied for 60 min, followed by thorough rinsing and drying (at room temperature with a household hair dryer). A second gel containing EEE-COONa and H_2_O_2_ was then applied for 30 to 240 min, during which localized polymerization occurred directly on the hair shaft (movie S2). Citric acid posttreatment restored conductivity to levels comparable to solution-processed coatings (fig. S15).

Polymerization remained confined to individual hair shafts, even when multiple hairs were simultaneously exposed to the monomer solution, allowing each strand to function as an independent conductive element (fig. S16). Alternatively, bundled hairs behaved as parallel conductors with decreasing resistance as the hair number increased (Fig. 3E). This strand-specific architecture enables precise control over electrode geometry and density: Single hairs define minimal recording sites, while bundles increase the effective electrode area and reduce impedance.

As with eDYE_C_, eDYE_P_ coatings exhibit mixed ionic-electronic conduction, with typical conductivity values of σ = 15 to 30 S·cm^−1^ and volumetric capacitance C* = 120 F·cm^−3^ (*52*). EIS performed on bundles comprising three coated hairs showed a substantial reduction in impedance compared to untreated hair (Fig. 3F). Single coated hairs exhibited |*Z*| ≈ 1 kΩ at 1 kHz. Impedance decreased with additional coating cycles, reflecting both reduced resistive losses and increased effective capacitance (Fig. 3G). Compared to eDYE_C_, eDYE_P_ coatings exhibited higher impedance, attributable to their lower intrinsic conductivity. This is further supported by impedance scaling with contact length, where shorter effective contact lengths result in lower impedance (fig. S17). CV measurements revealed a broad capacitive envelope that increased with the number of coated hairs, consistent with volumetric charge storage (fig. S18).

In summary, the eDYE_P_ process produces conductive hair with lower conductivity than eDYE_C_ but offers the distinct advantage of strand-specific, localized functionalization, enabling selective coating of individual hairs even within dense assemblies. The resulting coatings are mechanically robust and stable under bending (Fig. 3H) and retain functionality after washing, with conductivity recoverable through a single citric acid reconditioning step (Fig. 3I). This semipermanent behavior, maintaining electronic functionality during daily activities such as washing, sleeping, and physical movement, makes eDYE_P_ particularly well suited for extended or recurrent recordings over timescales of days to a week. However, prolonged mechanical abrasion can fully remove the coating (fig. S19).

Together, eDYE_C_ and eDYE_P_ define two complementary regimes of hair functionalization. eDYE_C_ provides highly conductive, conformal coatings that extend to the skin surface, enabling low-impedance interfaces suitable for immediate, high-performance recordings. In contrast, eDYE_P_ enables localized, strand-specific polymerization confined to the hair structure, supporting semipermanent functionalization with less skin coverage. This distinction enables tunable trade-offs between performance, localization, and operational lifetime.

### Transistors and amplifiers based on eHAIR

To further expand the functionality of eHAIR as a bioelectronic interface, we integrated conductive hair strands into OECT architectures, enabling active signal processing directly at the interface. OECTs operate at low voltages in aqueous environments and provide intrinsic signal amplification through volumetric ionic-electronic coupling, making them particularly suitable for electrophysiological signal transduction, substrate-free amplifier architectures, biosensing, and neuromorphic circuits (*54*–*56*). For device characterization, OECTs were configured with eHAIR as the channel and a Ag/AgCl electrode as the gate. In an alternative, fully hair-based configuration, two intersecting eHAIR strands defined the channel and gate, with an electrolyte applied at their junction (fig. S20). The output characteristics (*I*_D_-*V*_D_ at varying *V*_G_) displayed well-defined linear and saturation regimes (Fig. 4A), consistent with standard OECT operation (*57*). Positive gate voltages depleted hole carriers in the PEDOT:PSS-PDA–functionalized hair channel within the electrolyte-covered region, reducing the drain current (Fig. 4B and figs. S21 and S22), while negative gate biases restored channel conductivity. This reversible modulation confirms efficient ionic gating and stable electrochemical operation within the eHAIR channel. A representative eHAIR-based OECT (five coating cycles of PEDOT:PSS:DA), with a channel length of 1 mm and a width of ∼0.6 mm (diameter ∼0.2 mm), exhibited a maximum transconductance (*g*_m,max_) of 9.07 mS at *V*_G_ ≈ 0.2 V (Fig. 4B). This *g*_m,max_ value is comparable to planar PEDOT:PSS OECTs of similar dimensions (*46*), underscoring that the hair substrate does not compromise volumetric ionic-electronic charge modulation within the channel. The maximum transconductance increased systematically with the number of conductive coating cycles (Fig. 4C), reflecting increased channel thickness, higher volumetric capacitance, and reduced electronic resistance. These trends are consistent with established scaling laws in OECTs, where *g*_m_ ∝ μC*Wd/*L*, and demonstrate that coating cycles provide a direct handle over device performance.

**Fig. 4.**
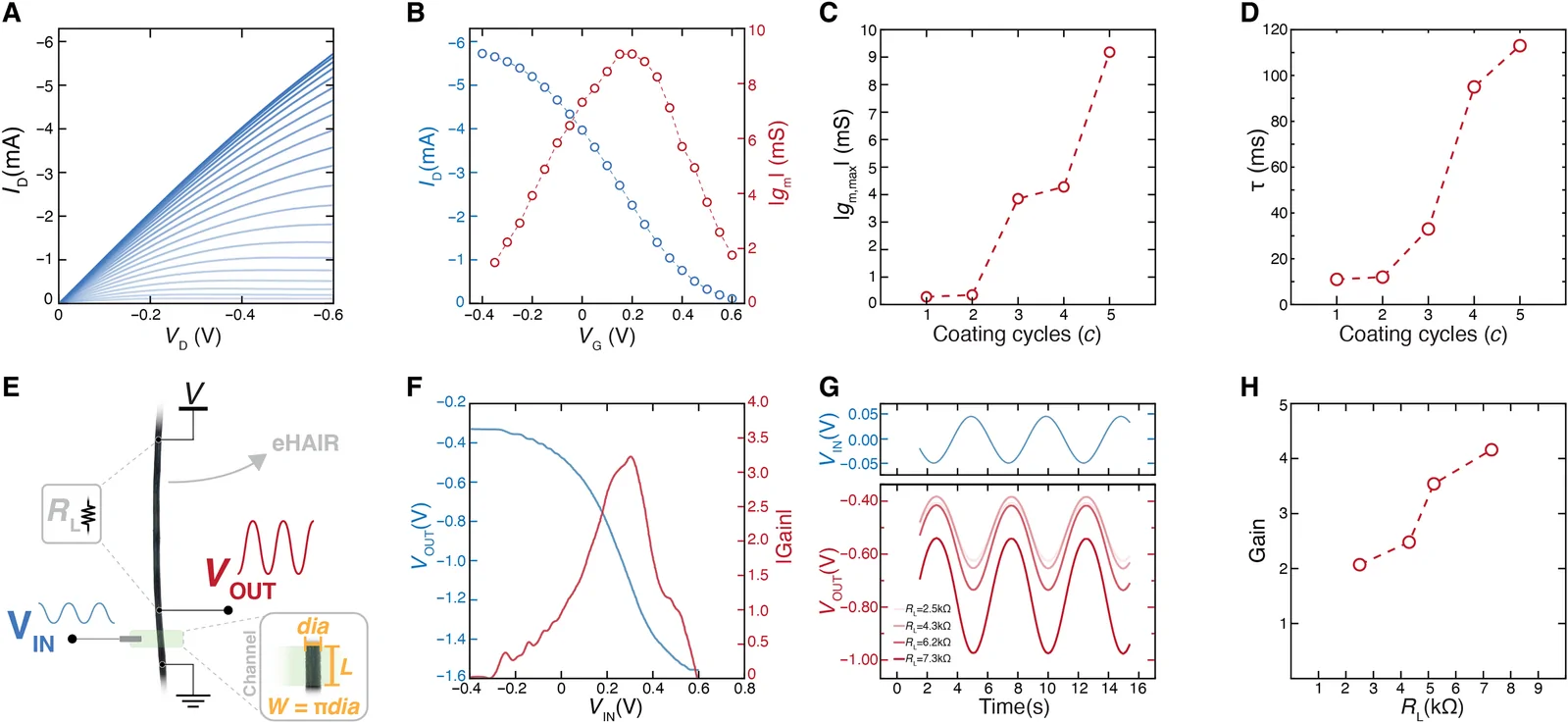
eHAIR-based OECTs and common-source amplifiers. (**A**) Output characteristics (*I*_D_-*V*_D_) of an eHAIR (*c* = 5 coating cycles) OECT at varying gate voltages ranging from −0.4 V (dark blue) to +0.6 V (light blue), showing clear linear and saturation regimes characteristic of OECT operation (*L* = 1 mm, *W* ≈ 0.6 mm). (**B**) Transfer and transconductance curves (*I*_D_ and *g*_m_) of the same device (for *V*_D_ = −0.6 V), demonstrating reversible gate-driven modulation of the channel current and a peak transconductance of 9.07 mS. (**C**) Maximum transconductance values (*g*_m,max_) of eHAIR OECTs as a function of the number of sequential eDYE_C_ coating cycles (1 to 5), showing a systematic increase in *g*_m,max_ with increasing coating cycles (*d* ≈ 0.01 mm after five coating cycles). (**D**) Temporal response (τ) of eHAIR OECTs as a function of the number of eDYE_C_ coating cycles (1 to 5). Thinner channels exhibit a faster response, while thicker coatings lead to slower ionic dynamics. (**E**) Schematic of the eHAIR-based common-source amplifier architecture, where the eHAIR (*c* = 5) OECT serves as the active transistor and the intrinsic line resistance of the hair functions as the load resistor (*R*_L_). (**F**) Small-signal voltage amplification for a fixed load resistance (*R*_L_ = 2.2 kΩ), showing a maximum voltage gain of ∼3 at an input voltage *V*_IN_ = 0.3 V. (**G**) Output voltage (*V*_OUT_) of the eHAIR common-source amplifier as a function of load resistance (*R*_L_ = 2.5 to 7.3 kΩ) for an input signal of 100 mV peak to peak (blue). Higher load resistance leads to increased amplification. (**H**) Voltage gain as a function of load resistance for the eHAIR common-source amplifier. The intrinsic line resistance of the hair acts as the load element, yielding a maximum gain of 4.16 at *R*_L_ = 7.3 kΩ.

Transient measurements further revealed that the effective time constant (τ) increased with coating thickness (Fig. 4D and fig. S23), indicating slower ionic charging and discharging dynamics in thicker channels. This behavior reflects the intrinsic gain-bandwidth trade-off of volumetrically gated devices: Thicker channels yield higher transconductance at the expense of temporal response (*58*). The observed τ values lie in the 10- to 100-ms range, corresponding to a frequency bandwidth of ∼10 to 100 Hz (even for relatively large device geometries; *W* ≈ 0.6 mm, *d* ≈ 0.01 mm, and *L* = 1 mm), which is well matched to electrophysiological signals such as EEG and electrocardiography (ECG).

Beyond device-level operation, eHAIR-based OECTs were configured as common-source amplifier units by leveraging the intrinsic resistance of the conductive hair segment as a built-in load resistor (*R*_L_) (Fig. 4E). In this architecture, *R*_L_ is defined solely by the length of the conductive hair between the drain and the output node, eliminating the need for discrete resistive components and enabling fully substrate-free amplifier circuits. The resulting transfer characteristics (Fig. 4F) showed clear voltage amplification, with the output voltage exhibiting an inverted response. Maximum small-signal gain was observed near the gate bias corresponding to peak transconductance, consistent with the relation |*A_v_*| ≈ *g*_m_*R*_L_ under saturation conditions, confirming proper biasing and amplifier operation. Dynamic measurements using sinusoidal input signals further demonstrated stable amplification behavior (Fig. 4G). The output amplitude increased systematically with increasing *R*_L_ while maintaining signal inversion and waveform fidelity, indicating that amplification is dominated by transistor transconductance rather than parasitic effects. Quantitatively, the voltage gain scaled monotonically with *R*_L_ (Fig. 4H), in agreement with the relation of small-signal voltage gain (*A_v_*) for common-source amplifiers. These results demonstrate that both gain and operating point can be set simply by adjusting the hair length, providing a unique, geometry-encoded tuning mechanism unavailable in conventional electronics.

Together, these results establish eHAIR as an active bioelectronic platform in which sensing, amplification, and interconnection are intrinsically integrated within the hair substrate itself. By combining volumetric ionic-electronic transduction with substrate-free circuit architectures, eHAIR-based OECT amplifiers enable ultracompliant, distributed, and scalable electrophysiological interfaces with built-in signal processing capabilities. A conceptual implementation for EEG recording is illustrated in fig. S24, in which adjacent conductive hairs or hair bundles are coupled through a gel electrolyte. In this configuration, one eHAIR interface serves as the gate electrode that senses the local EEG potential, while an adjacent eHAIR OECT acts as the active channel, locally amplifying the signal in a common-source amplifier configuration before transmission to the acquisition electronics.

### In vivo EEG recordings

As a proof of concept, to evaluate eHAIR performance under physiologically relevant conditions, we conducted EEG recordings in anesthetized rats using dyed native hair as the electrical interface (Fig. 5A and fig. S25). The eDYE process is also compatible with animal hair, enabling functionalization of native rat hair across length scales, from individual hairs to localized hair patches (figs. S26 and S27). For in vivo recordings, we used eDYE_C_ because of its rapid processing time, compatible with anesthesia constraints. Hair above the right visual cortex was dyed in situ using the eDYE_C_ formulation, while the contralateral hemisphere was recorded using gold (Au) electrodes mounted with a commercial EEG gel, while a similar EEG reference electrode was placed on the front of the rat head midsection between the two recording electrodes. Each interface had a contact area of ∼64 mm^2^. For eHAIR, no conductive gel was used; electrical contact was established directly through the dyed hair, with connections made at the proximal end of the hair bundle (figs. S7 and S25). In vivo EIS of the different interfaces, measured against an Au/EEG gel placed on the scalp (with hair removed), revealed consistently lower impedance for eHAIR compared to Au/EEG gel electrodes across the physiological frequency range (Fig. 5B). Low-pass-filtered (100 Hz; 50-Hz notch) EEG traces over a representative 2-s window showed larger recorded signal amplitudes for eHAIR compared to the Au/EEG gel electrode (Fig. 5C, top), consistent with improved electrode-skin coupling rather than differences in the underlying neural source. Time-frequency analysis further showed consistently higher spectral power across the 0.1- to 50-Hz range for eHAIR, indicating enhanced sensitivity to both slow and midfrequency neural oscillations (Fig. 5C, bottom). This enhancement persisted throughout extended recordings, as reflected in the averaged power spectra and power ratios (Fig. 5G), where the eHAIR interface exhibits a pronounced power gain relative to Au/EEG gel electrodes, reaching ∼3× to 4× in the low-frequency range (∼0.1 to 8 Hz) and remaining consistently above unity across the fully analyzed spectrum up to 50 Hz. The largest enhancement occurs in the low-frequency bands (δ, θ, and α), frequency ranges that are particularly sensitive to interface-skin impedance and motion-related artifacts (fig. S28) (*18*, *25*, *59*). The stronger gain below ∼15 Hz reflects the dominance of low-frequency physiological oscillations and the importance of low interface impedance in this regime. To distinguish physiological EEG activity from broadband recording noise, we additionally calculated a recording-derived signal-to-noise ratio (SNR) proxy from the preinjection recordings (fig. S29) using the high-frequency spectral noise estimate described in note S1. The median broadband noise amplitude spectral densities were comparable for eHAIR and Au/EEG gel (0.1620 and 0.1679 μV/√Hz, respectively), whereas the median SNR proxy was higher for eHAIR (17.86 versus 11.82 dB). Thus, the higher SNR proxy primarily reflected greater recorded EEG-band power rather than a lower estimated broadband noise floor. This enhanced EEG-band power is consistent with the lower electrode-skin impedance measured for eHAIR, which is expected to improve electrical coupling and reduce signal attenuation at the interface. The improved signal quality of eHAIR possibly arises from its intrinsically low electrode-skin impedance and efficient signal transmission through low-resistance hair shafts. In addition, the mechanical stability of hair, anchored within the scalp, reduces susceptibility to motion-induced artifacts. The enhanced interface performance can be attributed to two key factors. First, direct contact between the conducting polymer and the scalp eliminates the impedance contribution associated with conventional ionically conductive gels. Second, the high volumetric capacitance of conducting polymers increases the effective electrode area compared to metallic electrodes, improving charge coupling. A cross-electrode correlogram showed a clear diagonal structure with moderate correlation coefficients (up to *r* ≈ 0.5) and a minor asymmetry below the diagonal (Fig. 5H), indicating that both interfaces capture similar neural dynamics, while eHAIR provides improved signal amplitude and noise characteristics.

**Fig. 5.**
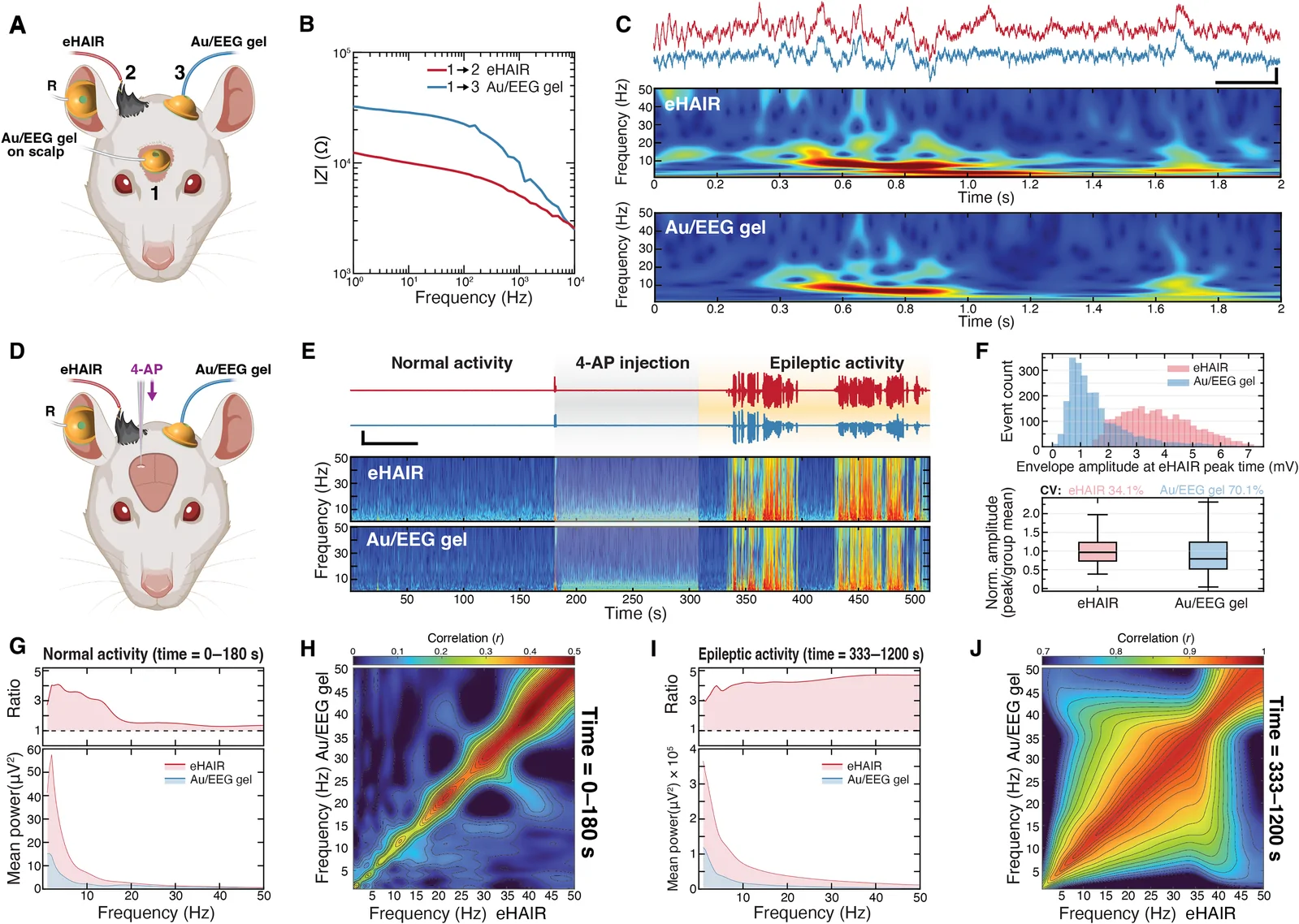
eHAIR enables low-impedance EEG interface and consistent detection of epileptic activity in vivo. (**A**) Schematic of the in vivo rat EEG configuration. eHAIR above the right visual cortex (interface area: ∼64 mm^2^; *c* = 5 coating cycles) and Au/EEG gel electrode at the contralateral site (interface area: ∼64 mm^2^). Reference Au/EEG gel electrode placed on scalp (without hair) for EIS in (B). (**B**) In vivo EIS spectra of eHAIR (1 → 2) and Au/EEG gel (1 → 3) electrodes measured against the Au/EEG reference. (**C**) Representative EEG traces (top; scale bar, 20 μV; 200 ms; eHAIR: red color; Au/EEG gel electrodes: blue color) and time-frequency spectrograms (bottom) for eHAIR and Au/EEG gel electrode over a 2-s time window. The EEG traces were measured against R (reference on right ear). (**D**) Experimental configuration for chemically induced seizures using intracortical injection of 4-AP. (**E**) Continuous EEG recording showing baseline activity, onset of epileptiform discharges following 4-AP injection, and sustained seizure activity. Scale bar, 5000 μV, 50 s (top). Time-frequency spectrograms reveal stronger and more broadband spectral signatures recorded by eHAIR compared to Au/EEG gel throughout epoch. Warmer colors represent higher relative power in logarithmic scale (bottom). (**F**) Distribution of the signal-envelope amplitudes recorded simultaneously by eHAIR and Au/EEG gel at the times of epileptiform peaks detected by eHAIR (top). Normalized peak-amplitude distributions (bottom; peak amplitude divided by the corresponding group mean) show lower event-to-event variability for eHAIR (CV: 34.1%) than for Au/EEG gel (CV: 70.1%), indicating more consistent detection of epileptiform events. (**G**) Mean power spectra and corresponding power ratios (eHAIR versus Au/EEG gel) during baseline activity. (**H**) Frequency-frequency correlation matrix between eHAIR and Au/EEG gel during baseline activity. (**I**) Mean power spectra and corresponding power ratios (eHAIR versus Au/EEG gel) during seizure activity. (**J**) Frequency-frequency correlation matrix during seizures. Panels (A) and (D) were partially created in BioRender. Spyropoulos, G. (2026) https://BioRender.com/b1lkrih.

We next evaluated performance during pathological activity by inducing epileptic seizures via intracortical injection of 4-aminopyridine (4-AP) (Fig. 5D). Following 4-AP administration, both interfaces detected the onset of epileptiform discharges; however, eHAIR recordings exhibited higher signal amplitudes throughout the seizure activity (Fig. 5E). Time-frequency analysis revealed stronger and more sustained broadband power across all epochs for eHAIR compared to Au/EEG gel electrodes. A matched-event analysis was performed to compare the amplitudes recorded simultaneously by the two interfaces at epileptiform peaks detected from the eHAIR signal (Fig. 5F). The absolute-amplitude distributions showed higher values for eHAIR than for Au/EEG gel (Fig. 5F, top). However, because the 4-AP injection was performed in the same hemisphere as the eHAIR interface, part of this amplitude difference may reflect proximity to the epileptogenic source. To assess recording consistency independently of the mean amplitude difference, peak amplitudes were normalized to the corresponding group mean (Fig. 5F, bottom). The normalized distributions revealed substantially lower event-to-event variability for eHAIR than for Au/EEG gel, as quantified by the coefficient of variation, a measure of variability relative to the mean (CV: 34.1% versus 70.1%; see note S2). This indicates a more consistent representation of temporally matched epileptiform events. Mean power spectra and power ratios during seizure epoch further showed elevated power for eHAIR across the fully analyzed frequency range (3× to 5×) (Fig. 5I). In contrast to baseline activity, seizure recordings exhibit broadband signal enhancement across the full frequency range. This reflects the high-amplitude, broadband nature of epileptiform activity, which reduces the relative influence of interface-limited attenuation and results in more uniform power gains across frequencies. During seizures, cross-electrode correlograms exhibited high diagonal correlations (*r* ≈ 0.7 to 1), indicating near-identical frequency-specific seizure dynamics across interfaces despite differences in signal fidelity (Fig. 5J). Although the two recording interfaces were positioned on contralateral hemispheres, several independent observations indicate that the improved recording performance primarily originates from the electrode-skin interface rather than differences in the underlying neural sources. Specifically, eHAIR consistently exhibited lower interface impedance and higher signal amplitudes and spectral power during both baseline and seizure activity, while the strong cross-correlation between recordings demonstrates that both interfaces captured the same underlying neural dynamics despite their different recording locations. To further demonstrate generality, we performed proof-of-concept recordings in humans using extracted eHAIR coated ex situ, capturing both EEG and ECG signals with clear physiological features, including alpha oscillations and well-defined cardiac waveforms (fig. S30). These results demonstrate that eHAIR functions as both an effective electrode and interconnect, with lower interface impedance and enhanced recorded signal amplitude, spectral power, and event-to-event consistency relative to conventional Au/EEG gel electrodes, consistent with the interface properties described above. Together, these properties enable enhanced EEG recordings with improved sensitivity, stability, and robustness under both physiological and pathological conditions.

## DISCUSSION

This work introduces a new paradigm in bioelectronics by transforming hair into an active, mixed-conducting interface for electrophysiology. By integrating conducting polymers with the intrinsic architecture of hair, we establish eHAIR as a substrate-free, biointegrated platform that combines sensing, interconnection, and signal amplification within a naturally occurring structure. Rather than designing increasingly sophisticated electrodes to overcome the presence of hair, eHAIR redefines hair itself as the bioelectronic interface. Compared with conventional dry electrodes, this approach eliminates the need for rigid conductive structures to mechanically penetrate dense hair. Compared with gel-based electrodes, the conductive interface is integrated directly onto the hair, removing the need for bulky gel reservoirs or conductive elements at the recording site. Unlike emerging in situ gelation or injectable bioelectronic interfaces that generate conductive materials on the skin surface, eHAIR localizes electrical functionality to the hair itself, preserving its natural flexibility and geometry while enabling both passive electrophysiological recording and active organic bioelectronics. The two complementary strategies, conformal coating (eDYE_C_) and localized in situ polymerization (eDYE_P_), enable tunable interfaces ranging from temporary, washable configurations to semipermanent, strand-specific functionalization. Both approaches preserve the skin interface, either forming seamless, low-impedance contact at the surface or remaining confined to the hair structure. Beyond passive electrodes, eHAIR supports active functionality through OECTs, including geometry-defined amplification and substrate-free circuit architectures. In vivo EEG recordings demonstrate improved signal amplitude, spectral power, and SNR compared to conventional gel-based electrodes, highlighting the advantages of direct, stable coupling. More broadly, this approach shifts bioelectronic design toward functionalizing native biological structures. Although natural hair growth may gradually shift the conductive region away from the scalp during prolonged use, eHAIR is intrinsically renewable. The conductive interface can be periodically regenerated by reapplying the coating or repeating the polymerization process, restoring low-impedance electrical contact as the hair grows. By using application methods equivalent to routine household hair coloring, eHAIR can be applied outside specialized facilities. Combined with electrical functionality directly within hair, eHAIR opens new opportunities for scalable, ultracompliant, and user-friendly interfaces for electrophysiology, brain-computer interfaces, and wearable health monitoring.

## MATERIALS AND METHODS

### Materials

PEDOT:PSS (Clevios PH1000) was obtained from Heraeus. DA-HCl, copper(II) sulfate pentahydrate (CuSO_4_·5H_2_O), hydrogen peroxide (H_2_O_2_), GOPS, 4-dodecyl benzene sulfonic acid, and ethylene glycol were purchased from Sigma-Aldrich. Tris(hydroxymethyl) aminomethane (tris) buffer was prepared at pH 8.5. Deionized water was used for dilution and cleaning purposes. All chemicals were of analytical grade and used without further purification. Natural hair was purchased from Amazon.de or Amazon.se, and anonymous volunteers donated hair for testing on different hair types.

All chemicals were purchased from commercial suppliers and used as received. EEE-COONa (*39*), ETE-COONa [sodium 2-(2-(2,5-bis(2,3-dihydrothieno[3,4-b][1,4]dioxin-5-yl)thiophen-3-yl)ethoxy)acetate] (*17*), ETE-PC [2-(2,5-bis(2,3-dihydrothieno[3,4-*b*][1,4]dioxin-5-yl)thiophen-3-yl)ethyl (2-(trimethylammonio)ethyl)phosphonate] (*35*), and ETE-S [sodium 4-(2-(2,5-bis(2,3-dihydrothieno[3,4-b][1,4]dioxin-5-yl)thiophen-3-yl)ethoxy)butane-1-sulfonate] (*60*) were synthesized according to previously reported methods.

### Method 1: eDYE_C_ formulation

The PDA coating on hair was obtained by two methods. The first method was via oxidative self-polymerization of dopamine under alkaline conditions. DA-HCl was dissolved in 10 mM tris buffer (pH 8.5) to a final concentration of 2 mg/ml. Clean hair samples were immersed in the freshly prepared dopamine solution and left undisturbed at room temperature for 6 to 12 hours to allow in situ polymerization and PDA deposition. The hair samples were then removed and gently rinsed with deionized water to remove unreacted dopamine and loosely adhered PDA particles. Last, the PDA-coated hairs were dried using a nitrogen gun (*32*, *33*). The second method was by rapid, CuSO_4_/H_2_O_2_–catalyzed in situ polymerization of dopamine under mildly alkaline conditions. DA-HCl was dissolved in 10 mM tris buffer (pH 8.5) to a final concentration of 1 mg/ml. CuSO_4_ and H_2_O_2_ were then added to final concentrations of 10 and 15 mM, respectively (pH maintained at 8.5). Cleaned hair samples were immediately immersed in the reaction solution. Polymerization and PDA deposition started upon addition of CuSO_4_/H_2_O_2_ and proceeded for 5 min. The PDA-coated hair was then removed and rinsed thoroughly with deionized water and dried using nitrogen gun. (*28*, *30*, *31*). To obtain the eDYE solution, the DA-HCl solid was added directly to a PEDOT:PSS (1 wt %) solution at a fixed concentration of 10 mg of DA-HCl per 10 ml of PEDOT:PSS solution. The mixture was stirred continuously for 30 min to ensure complete dissolution of DA-HCl and thorough interaction with PEDOT:PSS (*34*). In the dopamine-doped PEDOT:PSS solution, we added GOPS at a concentration of 1 vol % to act as a cross-linker. Ethylene glycol was added at a concentration of 20 vol %, and one or two drops of 4-dodecyl benzene sulfonic acid were added. After the addition of all these components, the solution was stirred continuously for few minutes to ensure a homogeneous mixture (*61*, *62*).

The eDYE solution was applied over the PDA-coated hair using a micropipette by dispensing droplets along the length of the hair to promote uniform coverage followed by drying with a hair dryer at a temperature of 30° to 35°C for 7 to 9 min. We repeated this deposition-drying cycle four or five times to obtain uniform and thicker eDYE coating.

### Method 2: eDYE_P_ formulation

For method 2, hair strands were bundled at a single end using tape to keep the multiple fibers aligned. All strands were thoroughly cleaned by using only soap and water and then dried completely with a nitrogen gun. Then, a 4-ml mixture was prepared in phosphate-buffered saline (PBS) containing the desired concentration of the enzyme and 2.5 mM EDC/sulfo-NHS. Formulations with other enzymes were prepared in the same way with the desired enzyme concentration according to the units. The hair strands were immersed in the solution so that the taped ends remain outside the vial while the full length of each strand was submerged. The vial was placed on a stirring plate at low RPM, and the strands were incubated for 1 hour. After incubation, the strands were sequentially soaked for 5 min in three separate beakers containing deionized water, with brief rinsing performed between transfers. The samples were then dried with nitrogen. Next, EEE-COONa with a H_2_O_2_ solution was prepared by dissolving EEE-COONa in 90% deionized water and 10% (v/v) dimethyl sulfoxide and then adding H_2_O_2_ at a molar concentration equal to half that of the monomer. Solutions with other monomers were prepared in the same way. The solution was stirred until it was clear. The hair strands were then immersed in the solution, with the taped ends kept outside the vial while stirring at low RPM. For time-course experiments, individual strands were sequentially removed from the vial at predefined time points. Hair was first dried with N_2_, then washed with di-water for 1 min, and then dried again; the mentioned coating process can be repeated for additional coatings on top of already formed ones to produce thicker coatings with higher conductance. Unless otherwise stated, samples used for characterization underwent one coating cycle. For multiple coatings before enzyme functionalization, the hair strands were first immersed in a 0.1% (w/w) poly-l-lysine solution. Poly-l-lysine can act as an adhesion promoter for enzyme functionalization on the underlying polymer coating. Citric acid (0.3 M) was applied at the hair following a drying step with a heat gun at 70° to 93°C for 2 min. The process was repeated for three times.

A similar process occurs with the gel formulation, in which the gel contained 60% (w/w) sorbitol and di-water for the enzyme. For the monomer solution, the gels contained 60% (w/w) sorbitol, 90% di-water, and 10% dimethyl sulfoxide as the supporting electrolyte components.

### Electrical insulation of eDYE-coated hair

A 10% (w/v) PVA solution was prepared by slowly dissolving 10 g of PVA in 100 ml of deionized water while stirring the mixture continuously at a temperature of 80° to 90°C. The solution was then allowed to cool to a temperature range of 40° to 50°C while maintaining continuous stirring. After cooling, GOPS was added to the PVA solution at a concentration of 1 wt %. The solution was stirred continuously for an additional 30 min at 40° to 50°C to ensure thorough mixing and initial reaction between GOPS and PVA.

The prepared PVA-GOPS solution was applied using a dropper or Pasteur pipette through the dropping method along the length of hair, followed by drying at a temperature of 80° to 90°C for 30 to 45 min. This resulted in a stable, cross-linked dielectric film over the eDYE-coated hair’s surface (*50*).

### Removal of eDYE_C_

The eDYE_C_ coating was removed using either a regular shampoo wash or a dry shampoo method. For the shampoo wash, the eDYE_C_-coated hair was washed with mild shampoo and gently massaged to form lather for 1 to 2 min. It was then rinsed thoroughly with deionized water and dried using a hair dryer. For the dry shampoo method, dry shampoo was sprayed evenly onto the dyed hair (from a distance of 15 to 20 cm). The hair was gently rubbed for 1 to 2 min, so the dry shampoo could absorb the dye residue. After that, the remaining product and loosened residue were removed by brushing.

### Electrochemical and electrical characterization

EIS measurements were performed in a conductive gel electrolyte (axion electrode contact gel) using a PalmSens4 over a frequency range of 1 Hz to 1 MHz, using two-electrode configuration; eDYE-coated hair strand electrodes were measured against a Ag/AgCl wire electrode, which served as a combined reference and counter electrode. CV was performed to measure capacitance using the same experimental setup over the range from −0.75 to 0.75 V.

For eDYE_P_, EIS measurements were performed in 1× PBS using a Gamry 1010B potentiostat over a frequency range of 20 kHz to 0.4 Hz (10 points per decade), with an ac perturbation amplitude of 10 mV and a dc bias applied versus a Ag/AgCl reference electrode. For the two-electrode configuration (bulk films), photopolymerized films (no prior water washing) on large-area Au electrodes were measured against a Ag/AgCl pellet, which served as a combined reference and counter. For OECTs, the channel acted as a working electrode through shorting source and drain. The data were fitted with a Randles equivalent circuit *R* − ((*R* − *W*)||CPE), where CPE is a constant phase element, and *W* stands for the Warburg impedance element. Measurements from three different channels were taken and the median taken for the capacitance. The capacitance was extracted from the constant phase element *C*_eq_ = *Q*^1/*n*^*R*^(1−*n*)/*n*^. Data analysis was performed by using Python 3.9 and QtiPlot. The schematics were prepared using Adobe Illustrator.

A constant voltage of 6 V was applied using a SIGLENT SPD1168X power supply to the eDYE hair, which was attached to the light-emitting diode (LED), causing the LED to successfully illuminate. The *I*-*V* characteristics were measured by a Keysight B2902A Precision Source/Measurement Unit.

eHAIR-based OECTs were electrically characterized using a Keysight B2902A Precision Source/Measurement Unit with 1× PBS as an electrolyte and Ag/AgCl as gate electrodes. For *I*-*V* characteristics, the drain voltage (*V*_D_) was applied as linear sweeps from 0 to −0.6 V, and constant voltages (*V*_G_) from −0.4 to 0.6 V were applied to the gate. For temporal-response measurements of eHAIR-based OECTs, constant *V*_D_ was applied at −0.4 V (saturation regime) (*58*, *63*, *64*). Data were analyzed with a custom Python or MATLAB script.

### Rat in vivo experiment

All procedures were conducted in accordance with the European Directive 2010/63/EU and were approved by the Animal Experimental Ethical Committee of Ghent University (ECD 22/39 and ECD 24/57). We used female Sprague-Dawley rats (weighing 150 g) aged 16 weeks. Anesthesia was induced using 5% isoflurane delivered in oxygen at 2 liters/min until the animal lost consciousness. Then, the hair was cleaned with soap (Hibiscrub) to remove the surface oils.

#### Recording of spontaneous activity

EEG signals were recorded using an eHAIR interface over the right visual cortex and an Au electrode with conductive gel (Axion Contact Gel) over the left visual cortex. Each recording interface had an area of approximately 64 mm^2^. Then, the eHAIR bundle was electrically connected to a single wire and functioned as a single recording interface. Au electrode was placed on the right ear and served as the reference electrodes, as shown in Fig. 5. Using Open Ephys Acquisition Board, we simultaneously recorded the spontaneous activity of the brain from Au and eHAIR electrode for 300 s under isoflurane anesthesia (2% in oxygen at a flow rate of 0.8 liters/min). Throughout the recording, the rat’s core body temperature was maintained at 37°C.

#### Seizure induction

Epileptic seizures were induced by intracortical injection of 4-AP (500 nl of 15 mM solution). The injection was delivered at a rate of 250 nl/min for a total injection time of 2 min.

#### Data analysis

Data were analyzed with a custom Python or MATLAB script.

### Human EEG and ECG recordings

All EEG and ECG recordings were conducted on a healthy volunteer. EEG signals were recorded using extracted hair strands coated ex situ with eDYE placed over the occipital region of the scalp with conductive gel applied to facilitate electrical coupling. TENS electrodes were positioned on the forehead and behind the ear to function as the reference and ground electrodes, respectively. During the recordings, the volunteer was instructed to alternately close the eyes, blink, and open the eyes for predefined time intervals. Similarly, for ECG measurements, ex situ eHAIR–coated extracted hair was placed on the chest with and without conductive gel. TENS electrodes were placed on the right wrist and lower leg, serving as the reference and ground electrodes, respectively. All recordings were acquired using an Open Ephys 512 channel Acquisition Board (*65*). The proof-of-concept human EEG and ECG recordings were performed as self-experiments by the first author using ex situ–coated extracted hair. The participant voluntarily took part in the study and provided written informed consent for the acquisition and publication of the data and images presented here. No in situ coating, invasive procedure, or intervention on the participant’s skin was performed.

### Ex vivo skin histology for eDYE formulations—Human skin sample preparation

Ex vivo skin histology was essential to confirm that eHAIR coatings localize on the hair shaft and extend into the follicular opening toward the root without damaging the surrounding skin or hair follicles, thereby validating both the biocompatibility and mechanism of the bioelectronic interface. To this end, we used two standardized and ready-to-use human skin models from Genoskin to predict the clinical response, one for each eDYE method. Both human skin samples were thoroughly cleaned by using only soap and water to remove excess fat tissue and any residues from the medium where the samples were stored. Then, they were dried completely with a nitrogen gun. Before proceeding to the implementation of either eDYE method, the hair and also the skin were bleached to modify the hair melanin and thus modify the hair color for the better visualization of the conducting polymer film formation. For this purpose, a commercially available bleaching product (L’Oréal Paris Préférence) was applied to both human skin samples and incubated for 30 to 40 min. After bleaching, mild soap washing was repeated. Half of each sample was left untreated and used as a control for comparison with the coated part of the samples. In both methods, skin samples were maintained immersed in their culture medium, with only the surface exposed to air, to prevent dehydration and preserve tissue viability.

#### eDYE_C_ method—Rapid version

Upon preparation completion, one skin sample was used for the application of the eDYE_C_ formulation. PDA deposition was first performed on half of the sample, followed by application of the eDYE solution onto the PDA-coated region, as described previously. The solution was applied using a micropipette, ensuring uniform spreading of droplets along the length of the hair. The deposition process was repeated to obtain uniform and thicker eDYE coating.

#### eDYE_P_ method—Gel version

Part of the second skin sample was intended for evaluation of the eDYE_P_ formulation. We administered with a micropipette tip the enzymatic gel formulation on it, which contained 36% (w/w) d-sorbitol in PBS and left for 1-hour incubation. Next, we applied the monomer gel solution, which also contained 36% (w/w) d-sorbitol but in di-water with an incubation time of 1 hour as well. Then, the skin was rinsed with di-water and left to dry under ambient conditions.

#### Fixation protocol

Skin biopsies were placed in 4% formaldehyde (Polysciences Europe GmbH, Hirschberg an der Bergstrasse, Germany), left for 3 hours at room temperature, and then stored overnight at 4°C. Following fixation, samples were transferred to 30% sucrose at 4°C until the samples sank, meaning that they were fully saturated.

#### Embedding

Each skin sample was cut in half, resulting in two half-moon–shaped pieces, one control and one experimental. The samples were then embedded in an optimal cutting temperature matrix (Histolab Products AB, Askim, Sweden) in cryomolds. This embedding was done by placing the samples in the cryomolds with the epidermal surface facing sideways, with the goal of getting longitudinal sections while sectioning, and filling it with optimal cutting temperature medium. The samples were then snap-frozen by being dropped in isopentane surrounded with dry ice to keep the temperature low.

#### Sectioning

Cryosectioning was performed using the Leica CM1950 Cryostat (TrioLab AB, Mölndal, Sweden). Sections were collected onto the glass slides. Two thicknesses were collected: 10 μm and 30 μm.

#### Staining

Eosin staining (Histolab Products AB, Askim, Sweden), dehydration in ethanol, and clearing with Histolab Clear (Histolab Products AB, Askim, Sweden) were performed using the Linear Stainer Leica ST4020 (Leica Biosystems Nussloch GmbH, Germany). Hematoxylin was omitted because the color is very similar to both eDYEs. Sections were mounted using a Pertex xylene–based mounting medium (Histolab Products AB, Askim, Sweden) and coverslipped.

#### Imaging

Bright-field microscopy with a 40×/0.95–numerical aperture objective and 0.1369 μm/pixel was performed with the Olympus VS200 slide scanner. A virtual *z*-stack with a semiautomatic focus field was used to find the best focus depth across the section. Because of eosin-only staining, the exposure was set to 64.97 μs and postprocessed in Fiji using the subtract background feature with a rolling ball radius of 160 to 400 pixels.

## Supplementary Materials

### The PDF file includes:

Notes S1 and S2 Figs. S1 to S30 Legends for movies S1 and S2
