# Supplementary material for: Electronic hair: Transforming hair into a scalable bioelectronic interface
Source: Sci Adv. 2026 Nov 11;12(46):eaei3615. doi: 10.1126/sciadv.aei3615 (PMC13753996; doi:10.1126/sciadv.aei3615)
Supplement: Supplementary file 1 [file sciadv.aei3615_sm.pdf]

Science Advances
AAAS

Supplementary Materials for

Electronic hair: Transforming hair into a scalable bioelectronic interface

Shweta Shweta *et al.*

Corresponding author: Xenofon Strakosas, xenofon.strakosas@liu.se;
George D. Spyropoulos, georgios.spyropoulos@ugent.be

Sci. Adv. **12**, eaei3615 (2026)
DOI: 10.1126/sciadv.aei3615

The PDF file includes:

Notes S1 and S2
Figs. S1 to S30
Legends for movies S1 and S2

Other Supplementary Material for this manuscript includes the following:

Movies S1 and S2

Supplementary Note 1.

Spectral noise and SNR proxy

Pre-injection spectral noise and SNR analysis

Spectral noise and signal-to-noise ratio (SNR) were evaluated during the first 180 s of the pre-injection recording to compare the eHAIR electrode with the Au/EEG gel electrode. Signals were analyzed at the native sampling frequency of 30 kHz and converted to microvolts using a scaling factor of 0.195 μV per bit. No digital notch filter was applied before spectral estimation, allowing the 50 Hz mains component and its harmonics to remain visible in the power spectral density (PSD).

Each channel was divided into 2 s windows with a 1 s step, resulting in 179 windows per channel. The mean was removed from each window, and a Hann taper was applied before calculating the one-sided periodogram. The PSD was normalized by the sampling frequency and the energy of the Hann window and expressed in $\mu V^2/Hz$. The 2 s window length provided a frequency resolution of 0.5 Hz. For visualization, the median PSD was calculated at each frequency across all windows, and the interquartile range was used to indicate variability.

For each window i, EEG-band power was calculated by integrating the PSD, $S_i(f)$, from 1 to 45 Hz:

$$P\_EEG,i = \int_1^{45} S_i(f)\, df.$$

The broadband noise floor was estimated from 150 to 400 Hz. Frequency bins within ±2 Hz of the 50 Hz harmonics at 150, 200, 250, 300, 350, and 400 Hz were excluded from the calculation. The remaining frequency bins were summarized using their median PSD, N_i, to reduce the influence of residual narrowband spectral peaks. Noise amplitude spectral density was calculated as the square root of N_i and expressed in $\mu V/\sqrt{Hz}$.

To compare the estimated noise density with the EEG-band power, N_i was projected across the 44 Hz width of the 1-45 Hz EEG band:

$$P\_noise,i = N_i \times 44\ Hz.$$

The window-level SNR proxy was then calculated as:

$$SNR\_proxy,i\ (dB) = 10 \log_{10}[P\_EEG,i\ /\ P\_noise,i].$$

This metric is reported as an SNR proxy because the broadband noise density estimated from 150–400 Hz was projected across the 1–45 Hz EEG bandwidth, rather than obtained from an independent phantom or shorted-input measurement. Window-level noise ASD and SNR-proxy values were summarized using boxplots. The center line represents the median, the box represents the interquartile range, the whiskers extend to 1.5 times the interquartile range, and points outside the whiskers are displayed individually. Because adjacent windows overlapped by 50%, the window-level distributions were used to describe temporal variability and were not considered fully independent observations (Fig. S29).

Supplementary Note 2.

eHAIR-triggered envelope analysis

Continuous electrophysiological data were analyzed using a custom Python pipeline. The Open Ephys binary recording contained 64 interleaved channels stored as little-endian signed 16-bit integers and sampled at 30 kHz. The eHAIR and conventional Au/EEG gel signals corresponded to channels 15 and 51, respectively. Digital values were converted to millivolts using a conversion

factor of 0.195 μV per bit. Analysis was restricted to the interval from 300.000 s to the end of the recording (1329.134 s), thereby excluding the initial 5 min.

Signal preprocessing and envelope calculation

Before downsampling, each channel was low-pass filtered at 200 Hz using a centered, 1,501-tap finite-impulse-response filter constructed with a Hamming window. The filtered signals were decimated by a factor of 60, yielding an analysis sampling rate of 500 Hz. Each signal was subsequently band-pass filtered from 1 to 45 Hz using a second-order Butterworth filter implemented in second-order-section form and applied bidirectionally to obtain zero-phase filtering. No 100-Hz notch filter was applied. The analytic representation of each band-pass-filtered signal was calculated using the Hilbert transform. The instantaneous envelope amplitude was defined as the magnitude of the analytic signal:

$$A(t) = |x(t) + i\mathcal{H}\{x(t)\}|,$$

where x(t) is the 1-45 Hz signal and $\mathcal{H}\{x(t)\}$ is its Hilbert transform. Envelope amplitudes were expressed in millivolts.

eHAIR-triggered event detection

Events were detected exclusively from the eHAIR envelope. The peak-prominence threshold was defined as 50% of the 95th percentile of the eHAIR envelope over the complete analysis interval. Percentiles were calculated using the Hazen convention. This procedure produced a prominence threshold of 1.413476 mV.

Local maxima exceeding this prominence criterion were detected using the find_peaks function from SciPy. No additional minimum peak-height or minimum interpeak-interval criterion was imposed. A total of 2,419 eHAIR envelope peaks were identified.

For every detected eHAIR peak, the eHAIR envelope amplitude and the Au/EEG gel envelope amplitude were sampled at the identical time index. The resulting measurements therefore constituted temporally matched electrode pairs conditioned on events detected by eHAIR. Because events were detected from the magnitude of the Hilbert envelope, the analysis was insensitive to the polarity of the underlying voltage deflections.

Amplitude and variability calculations

For each electrode, the mean envelope amplitude and sample standard deviation were calculated across the 2,419 matched events. Relative amplitude variability was quantified using the coefficient of variation:

$$CV = 100 \times s/\bar{x},$$

where s is the sample standard deviation calculated with $n - 1$ degrees of freedom and $\bar{x}$ is the mean matched-event amplitude.

Across all eHAIR-triggered events, the mean ± SD envelope amplitude was 3.777 ± 1.287 mV for eHAIR and 1.593 ± 1.117 mV for Au/EEG gel. The corresponding CV values were 34.1% and 70.1%, respectively.

For visualization of relative dispersion, individual event amplitudes were divided by the mean amplitude for the corresponding electrode. This normalization was used only for the box-plot visualization and did not affect the statistical analyses.

Block-level statistical analysis

To reduce temporal pseudoreplication, the continuous analysis interval was divided into consecutive, non-overlapping 60-s blocks. The final block contained the remaining 9.134 s of the recording. Blocks containing at least three eHAIR-triggered events were retained for statistical analysis; all 18 blocks met this criterion.

Within each block, the median matched-event amplitude and CV were calculated separately for eHAIR and Au/EEG gel. The block, rather than the individual detected peak, was treated as the inferential unit.

Electrode amplitudes were compared using a two-sided paired Wilcoxon signed-rank test applied to the paired block-level median amplitudes. The hypothesis that eHAIR exhibited lower relative variability than Au/EEG gel was tested using a one-sided paired Wilcoxon signed-rank test applied to the paired block-level CV values, with the alternative hypothesis:

$$\mathrm{CV}(\mathrm{eHAIR}) < \mathrm{CV}(\mathrm{Au/EEG\ gel}).$$

Wilcoxon probabilities were calculated using the normal approximation. The block-level median-amplitude comparison yielded $P = 1.96 \times 10^{-4}$, and the directional CV comparison yielded $P = 9.82 \times 10^{-5}$. Reported P values were not adjusted for multiple comparisons.

Supplementary Figures

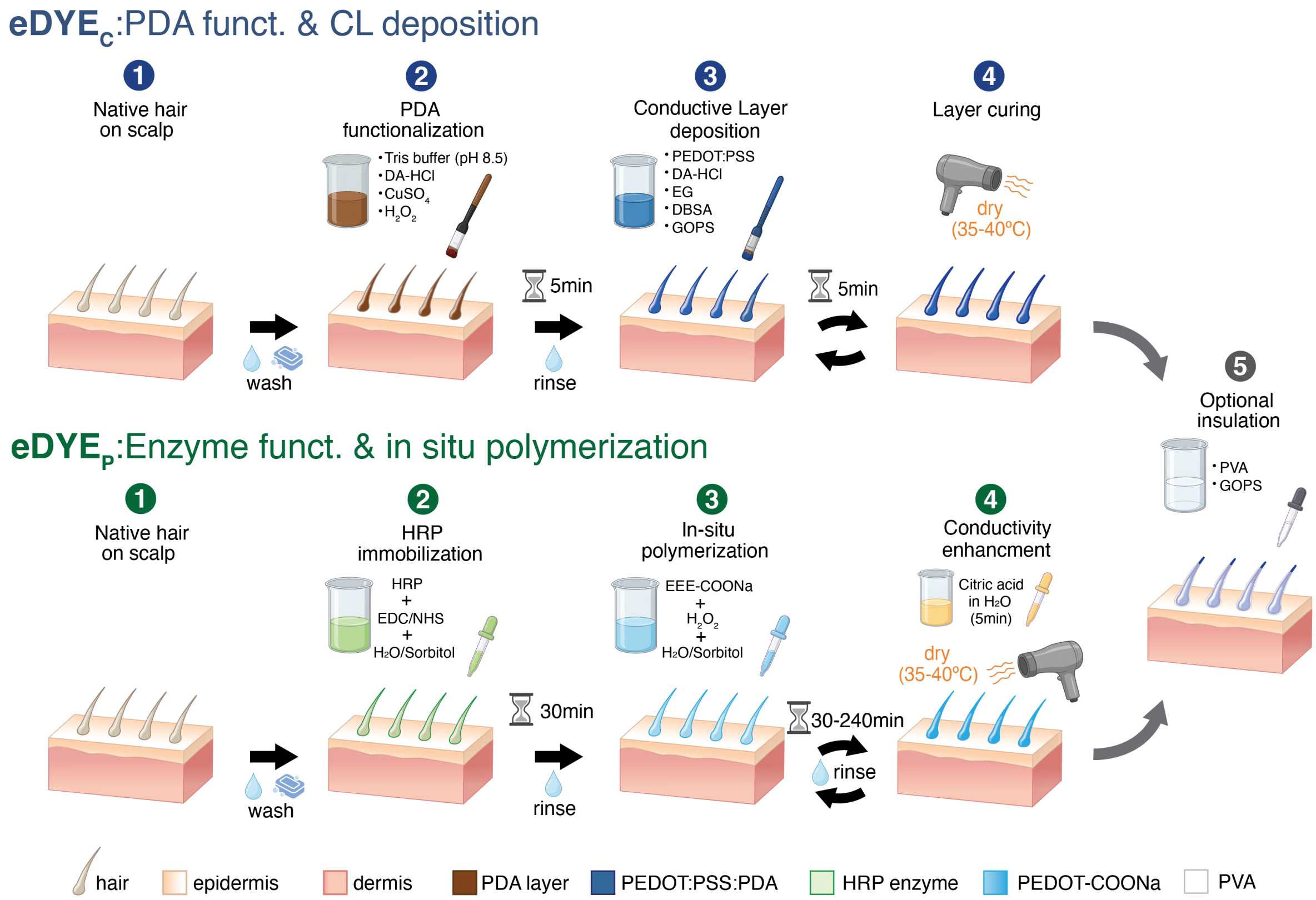

Fig. S1. Step-by-step schematic illustration of the in-situ coating steps for the two complementary eHAIR approaches. Top: eDYE$_C$ formation through rapid PDA functionalization of native hair, followed by deposition of a PEDOT:PSS-based conductive layer and mild thermal curing. Bottom: eDYE$_P$ formation through immobilization of HRP, followed by enzyme-assisted in situ polymerization of PEDOT-COONa on the hair surface and conductivity enhancement using a citric acid treatment. Both approaches are performed directly on hair while it remains anchored to the scalp. An optional PVA insulation layer can subsequently be applied to electrically isolate adjacent hair strands. Partially created in BioRender. Spyropoulos, G. (2026) https://BioRender.com/fdhsiaj.

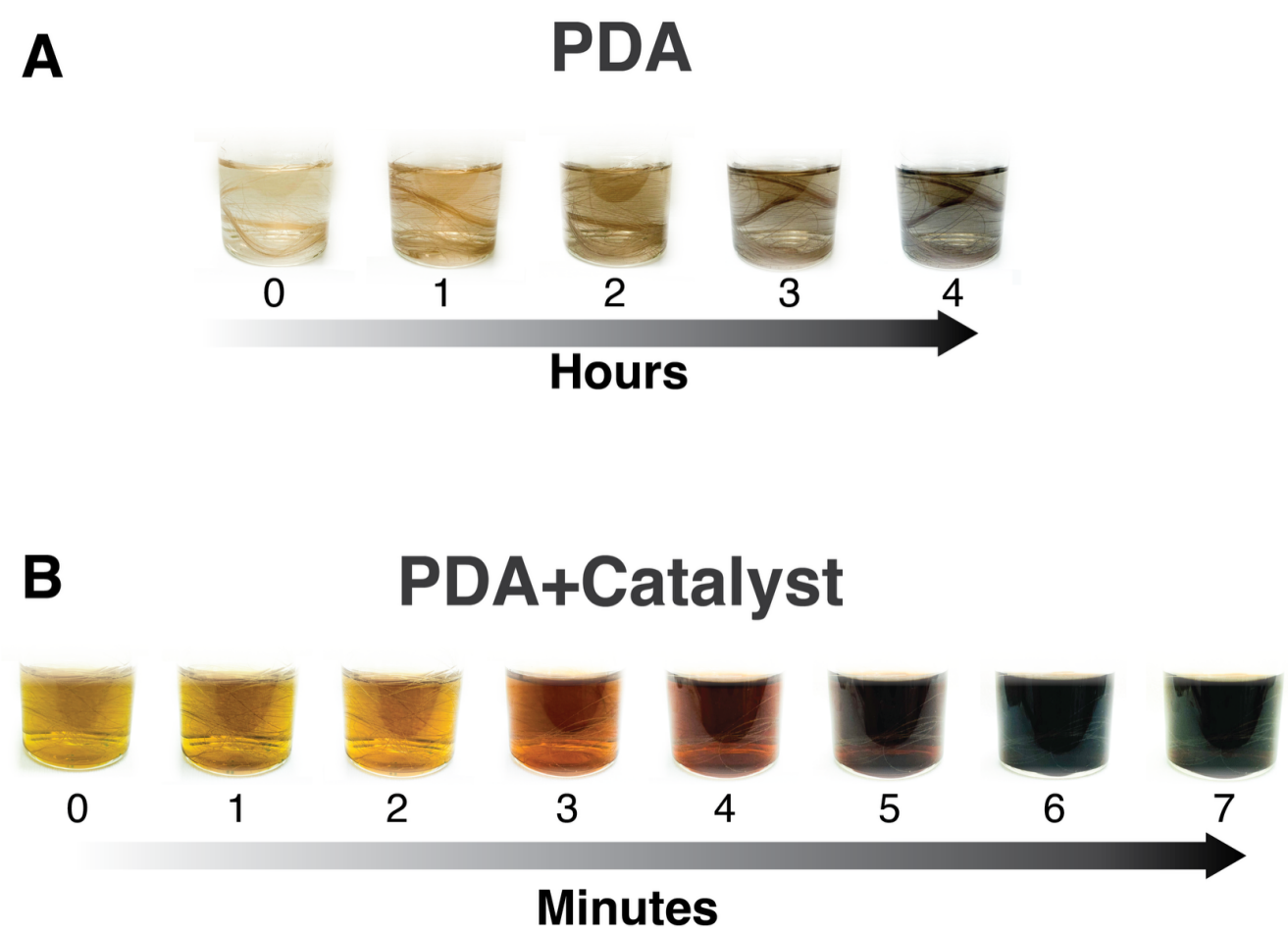

Fig. S2. **PDA functionalization of hairs. (A)** Gradual color change of the solution and hair strands (dipped into solution) over time (from 0 to 4 hours) indicates dopamine polymerization to polydopamine in Tris buffer (pH 8.5) without a catalyst. **(B)** Gradual color change of solution and hair strands (dipped into solution) over time (from 0 to 7 minutes) indicates dopamine polymerization to polydopamine in Tris buffer (pH 8.5) catalyzed by $CuSO_4$ and H_2O_2.

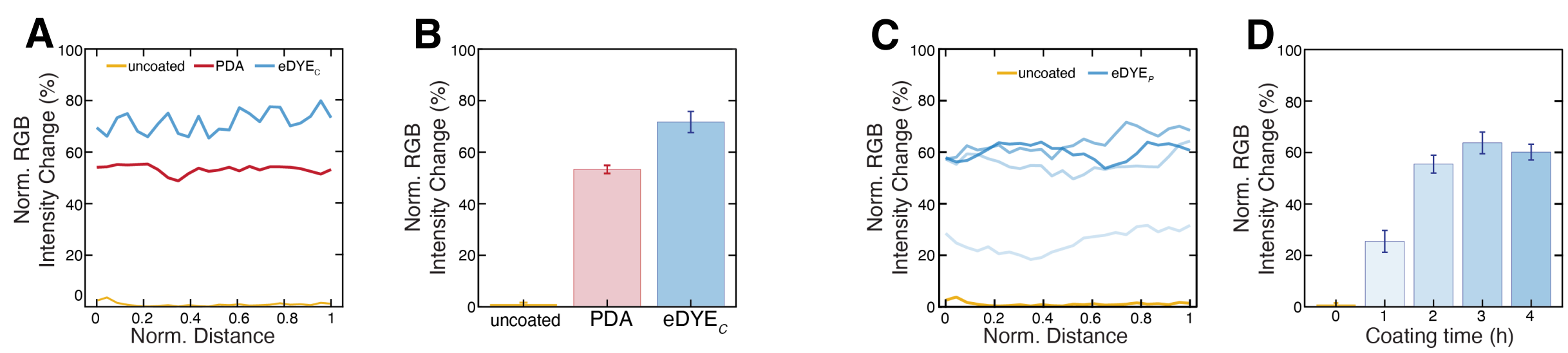

Fig. S3. Coating uniformity and growth dynamics of eDYEC on hair. (A, B) Normalized RGB intensity profiles along the hair length (normalized distance = 0.5cm) confirm enhanced contrast and longitudinal coating uniformity following PDA and $eDYE_C$ (c = 5 coating cycles) deposition. **(C, D)** normalized RGB intensity profiles and summary bar plots showing increasing coating density with polymerization time, with a plateau reached after approximately 2-3 hours.

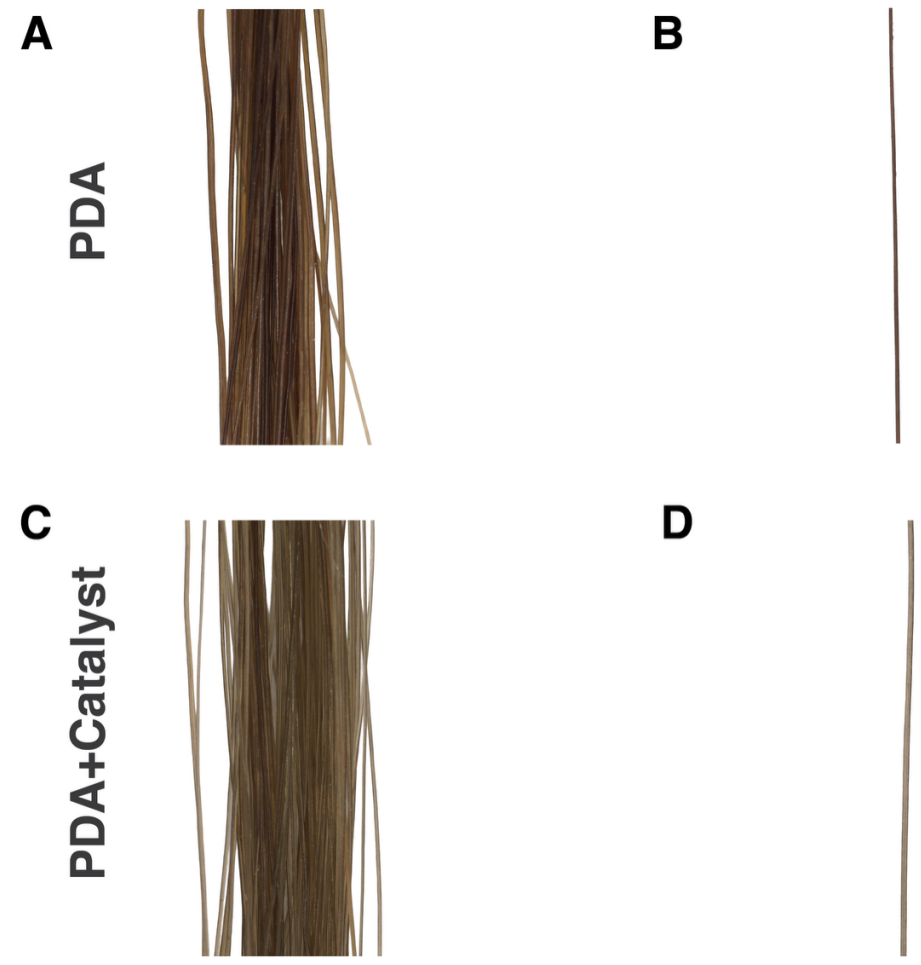

Fig. S4. Uniform PDA deposition on hair strands for both methods (SI 1). (A,B) Uncatalyzed dopamine polymerization in Tris buffer (pH 8.5). **(C,D)** Dopamine polymerization catalyzed with $CuSO_4$ and H_2O_2 in Tris buffer (pH 8.5).

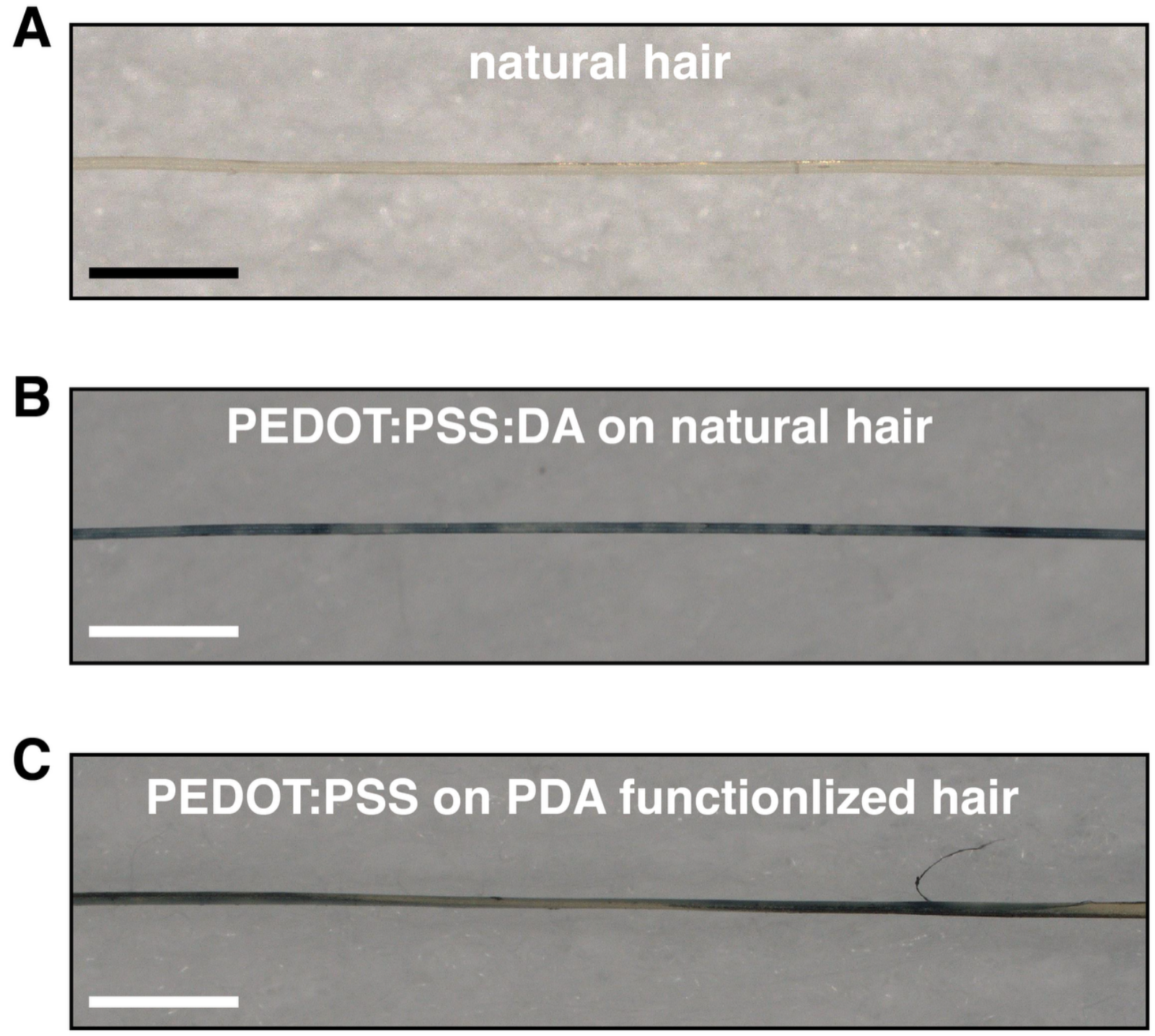

Fig. S5. Microscopic image of blonde hairs showing non-uniform coating due to incomplete ingredient formulation. (A) Natural blonde hair without any coating. **(B)** Edye coating on natural blonde hair, without pre-functionalization of hair with PDA. **(C)** Highly conductive PEDOT:PSS coating on the PDA-functionalized hair. Scale bar: 1 mm

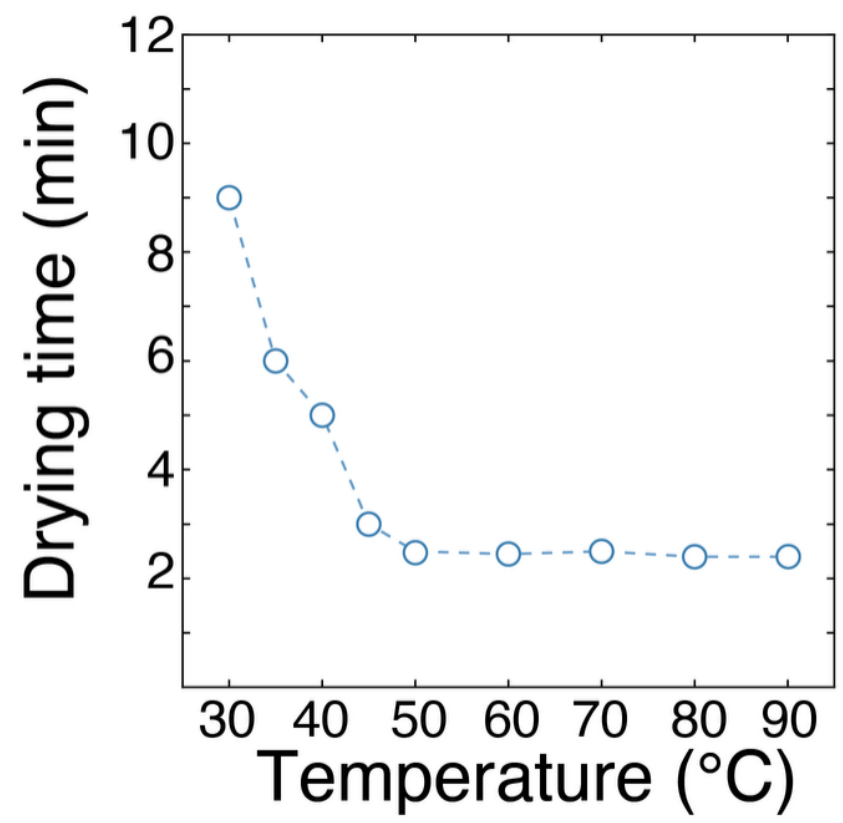

Fig. S6. **Rapid drying of eDYE$_c$ coating on PDA hair.** Drying time of the eDYE$_c$ coating (5 coating cycles) on the PDA-functionalized hair, by using hair dryer at different temperatures.

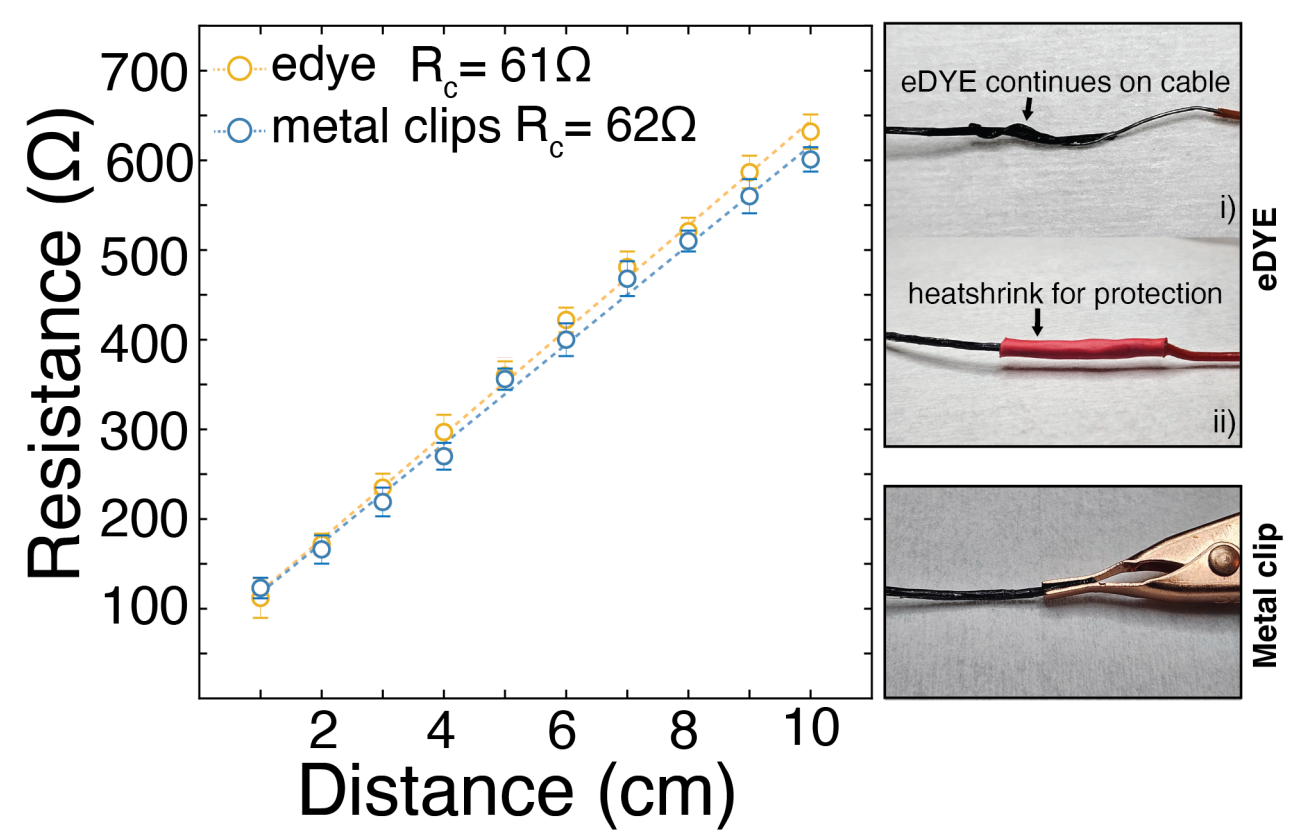

Fig. S7. **Stable electrical interfacing methods for eDYE$_c$ hair and the resistance as a function of distance**. Resistance variation as a function of distance for an eDYE$_c$ (c = 5 coating cycles) hair for different attachment methods used for electrical measurements: eDYE$_c$ hair integrated onto wire: (i) edye applied directly over overlapping area; (ii) edye applied with contact area protected by heat shrink, and through metal clip attachment. The contact resistance values remain nearly similar across both attachment configurations. The images illustrate the various attachment techniques used to connect hair samples.

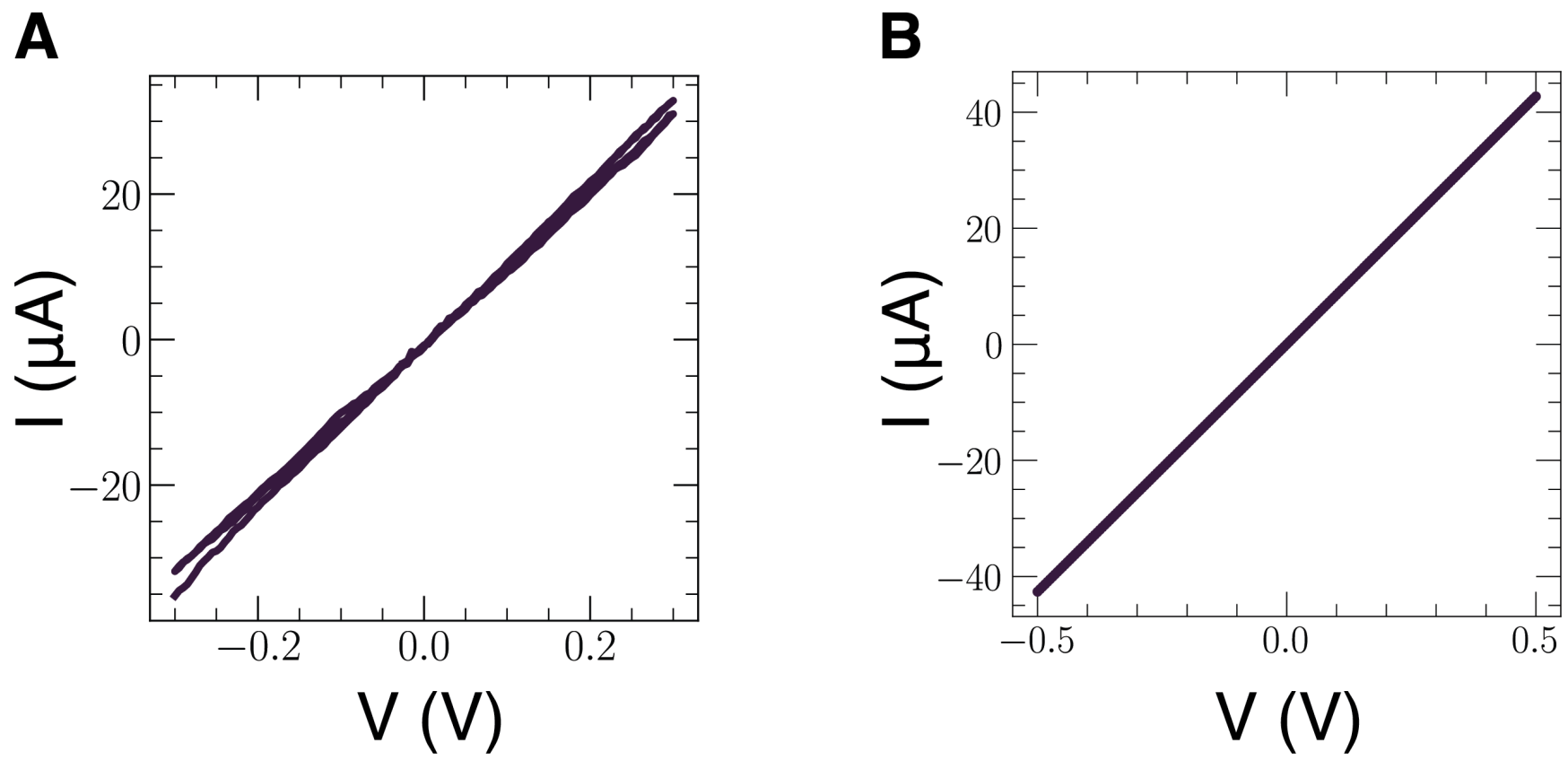

Fig. S8. In situ polymerization based on glucose oxidase: HRP and bilirubin oxidase. (A) IV in dry state from a blonde coated hair with gox/HRP (100 U/ml) and glucose 5 mM to fuel the enzymatic catalysis during 2 hours of polymerization. **(B)** IV in dry state from a blonde coated hair with Bilirubin oxidase (BOX) 8 U/ml and oxygen in solution during 24 hours of polymerization.

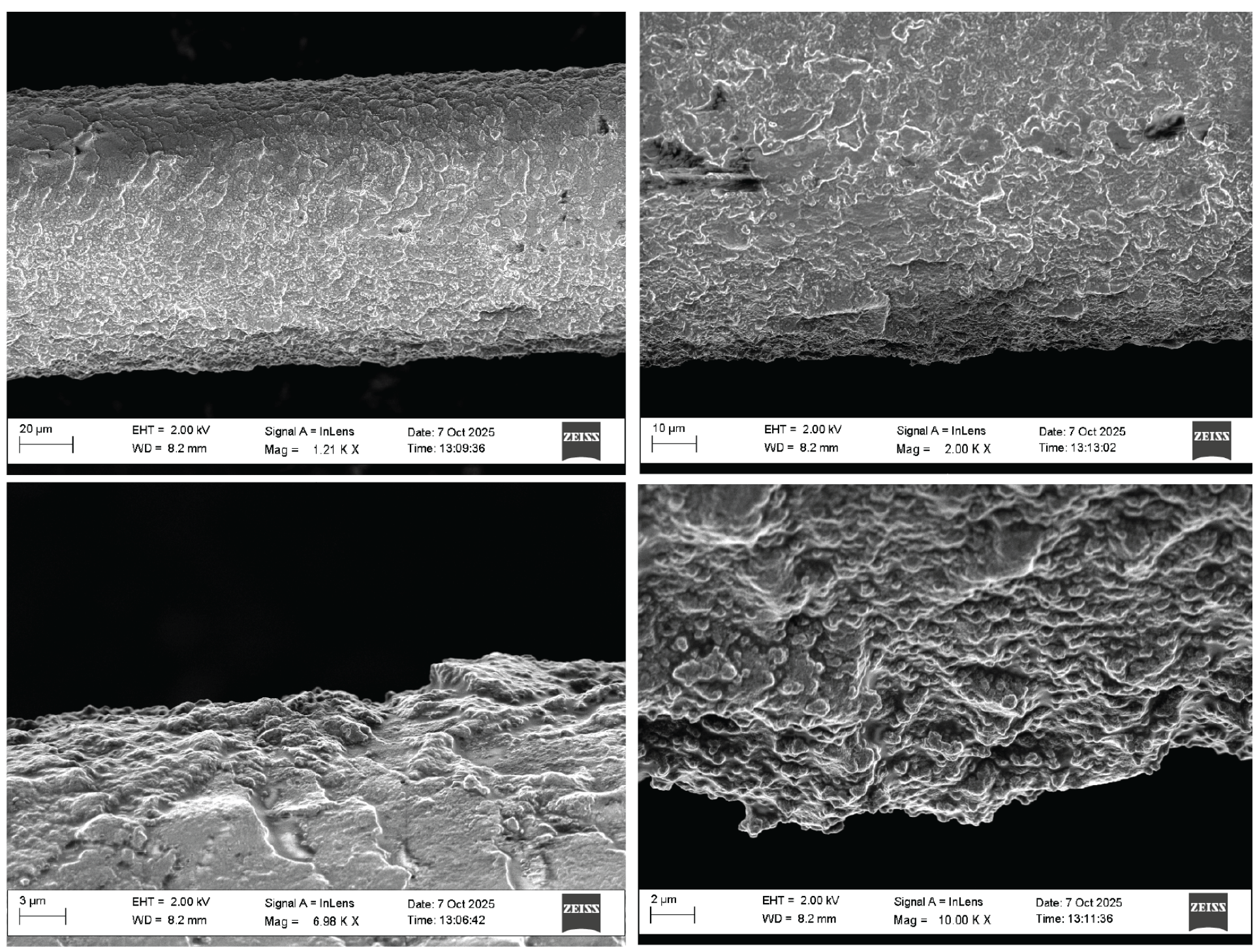

Fig. S9. SEM images show conductive coating with high surface area. Different magnifications of hairs are displayed to show the various levels of coating quality.

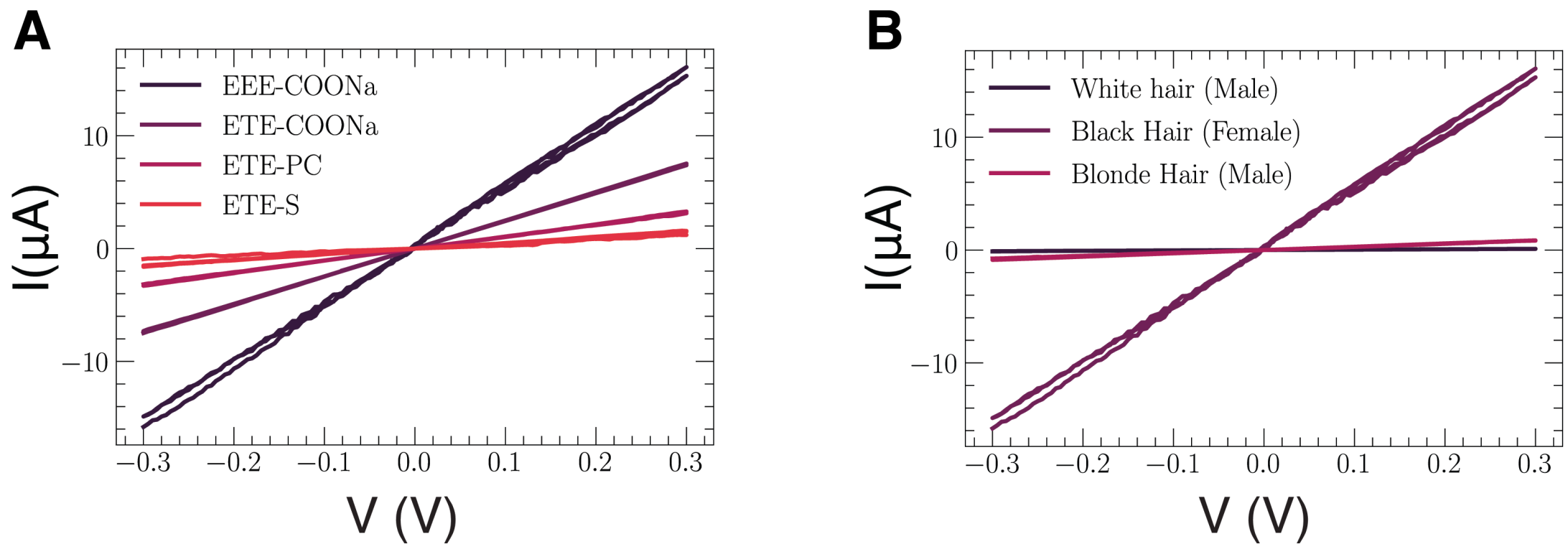

Fig. S10. In situ polymerization of different monomers showing EEE-COONa is the more conductive one. (A) IV in dry state from a blonde coated hair with gox/HRP and glucose to fuel the polymerization for coatings with different monomer analogs: ETE-COONa, ETE-PC, ETE-S. **(B)** IV for various coated hair. The hair were donated from healthy volunteers. The coating occurred with gox/HRP 250 U/ml and glucose and EEE-COONa 1mg/ml.

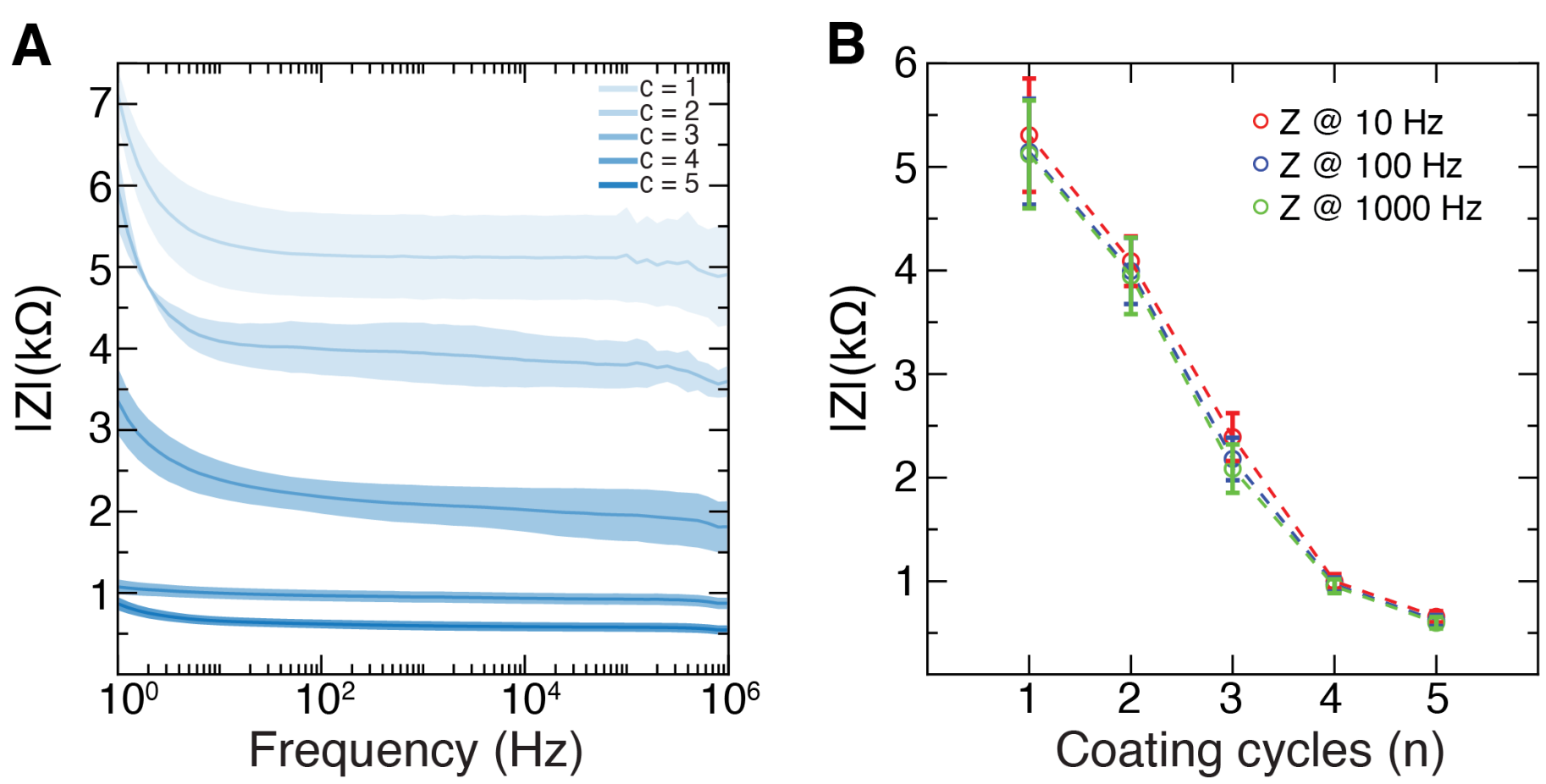

Fig. S11. Evolution of the impedance of individual eDYE$_C$-coated hairs with successive coating cycles.

(A) Electrochemical impedance spectra of individual eDYE$_C$-coated hairs after one (light blue) to five coating cycles (dark blue). Solid lines represent the mean impedance magnitude and shaded area represents ± SD (n = 5). **(B)** Impedance magnitude at 10, 100, and 1000 Hz as a function of the number of sequential coating cycles. The impedance decreases progressively with successive coating cycles and approaches a plateau after approximately four to five cycles. Data are presented as mean ± SD (n = 5).

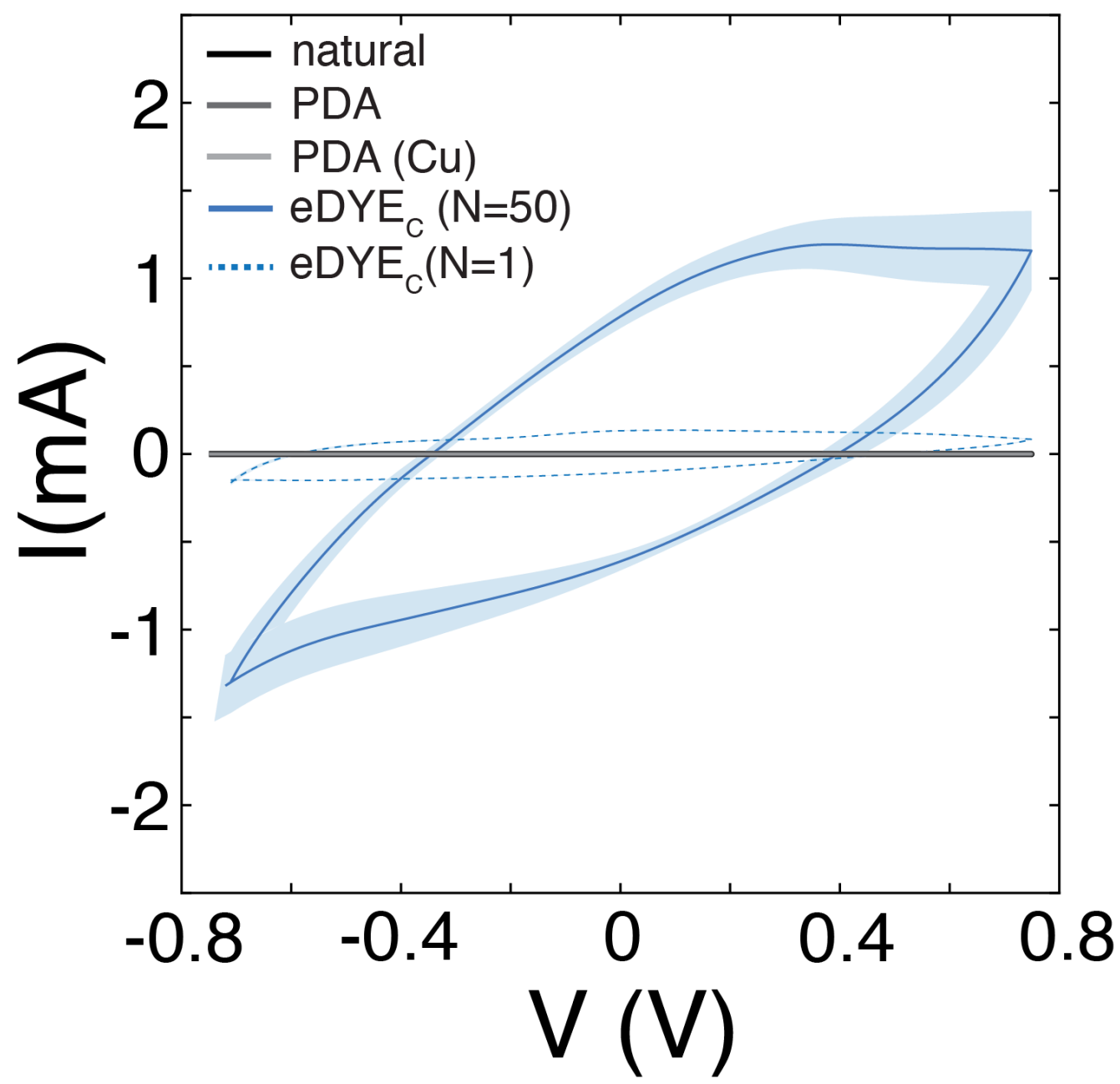

Fig. S12. EDYE$_C$ hair shows capacitive behavior characteristics. Cyclic voltammograms of eDYE$_C$ (c = 5 coating cycles) single-hair (N = 1) and fifty-hair (N = 50) electrodes, showing capacitive, mixed-conducting behavior characteristic of PEDOT-like OMIECs.

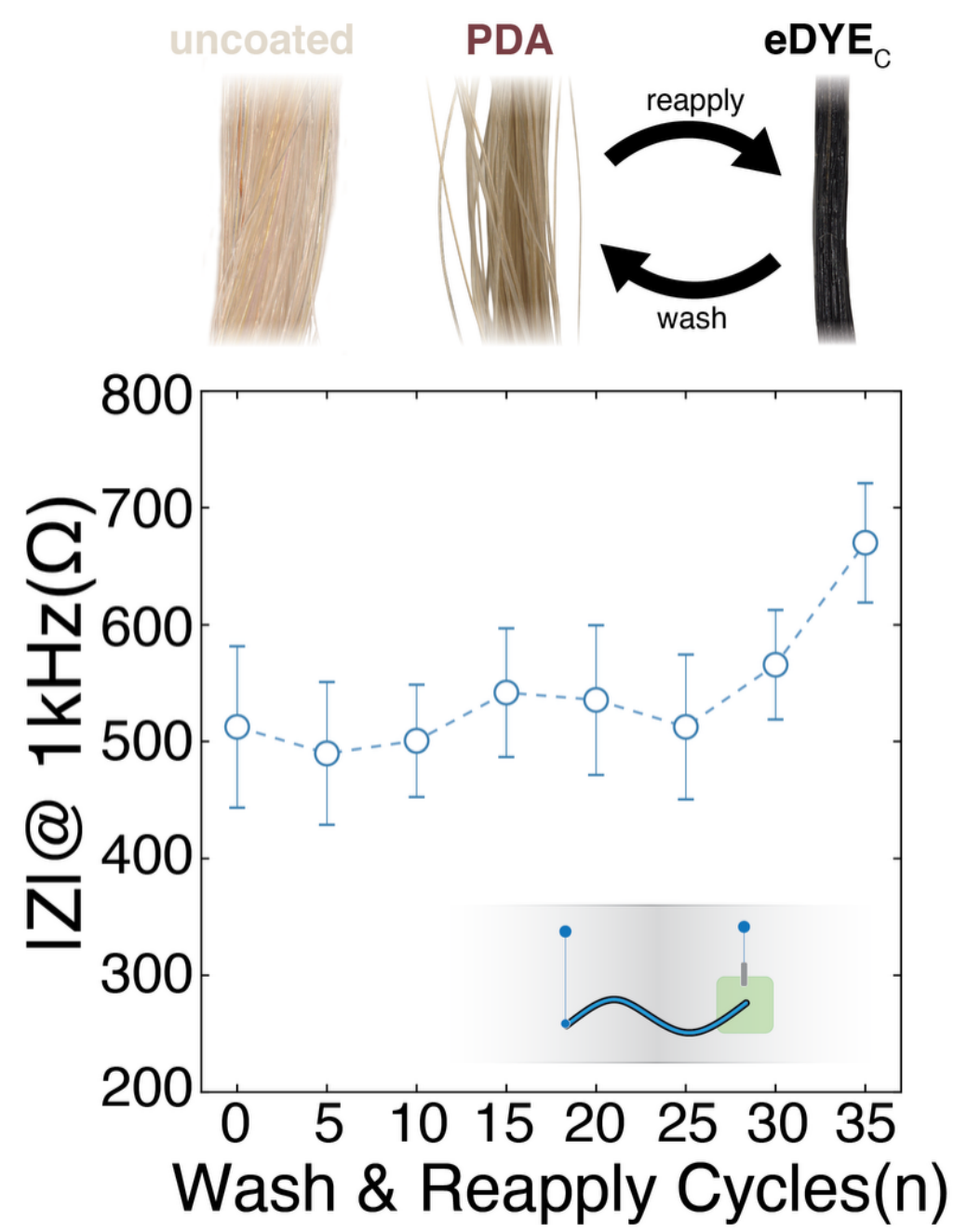

Fig. S13. Wash-Reapply cycle for eDYE coating. Impedance at 1 kHz for an eDYE$_C$ (c = 5 coating cycles) hair after washing and reapplication of eDYE coating on PDA-functionalized hair showing consistent values up to 25 cycles.

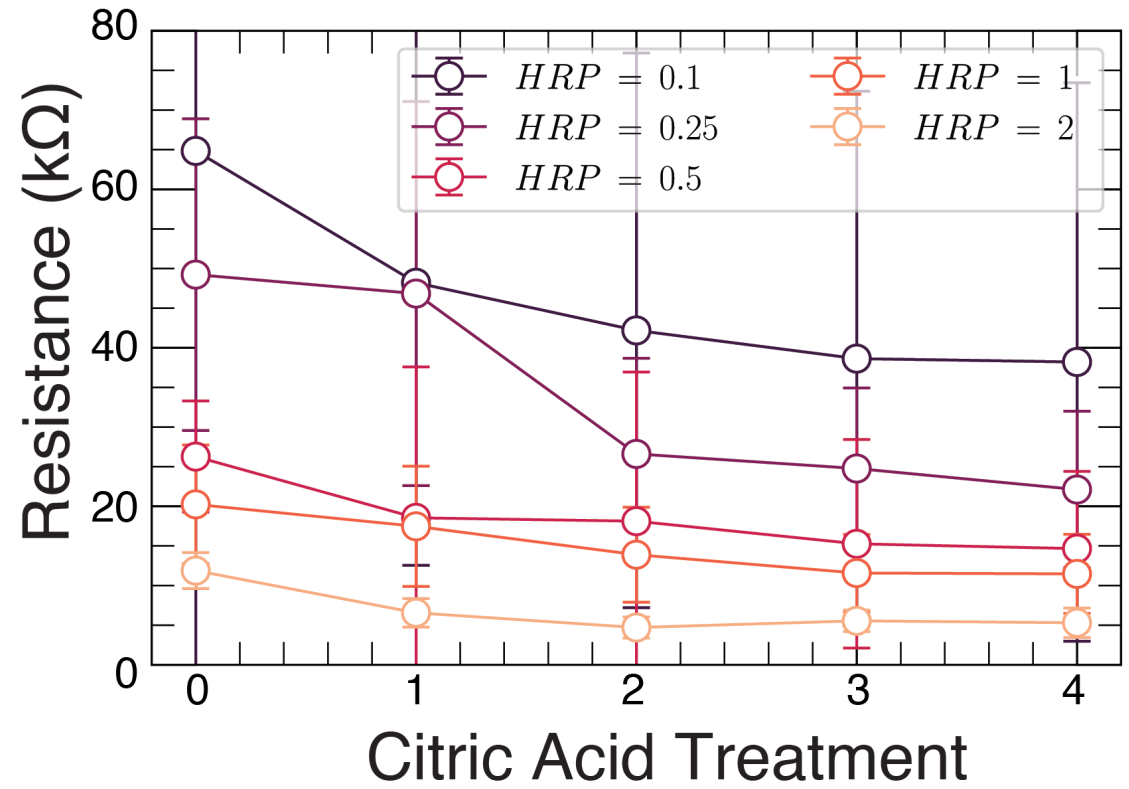

Fig. S14. Conductivity improves with subsequent citric acid treatments. Resistance measurements vs number of citric acid treatments on coatings with different enzyme concentrations in mg/ml (107.4 U/mg activity). (c = 1 coating cycle).

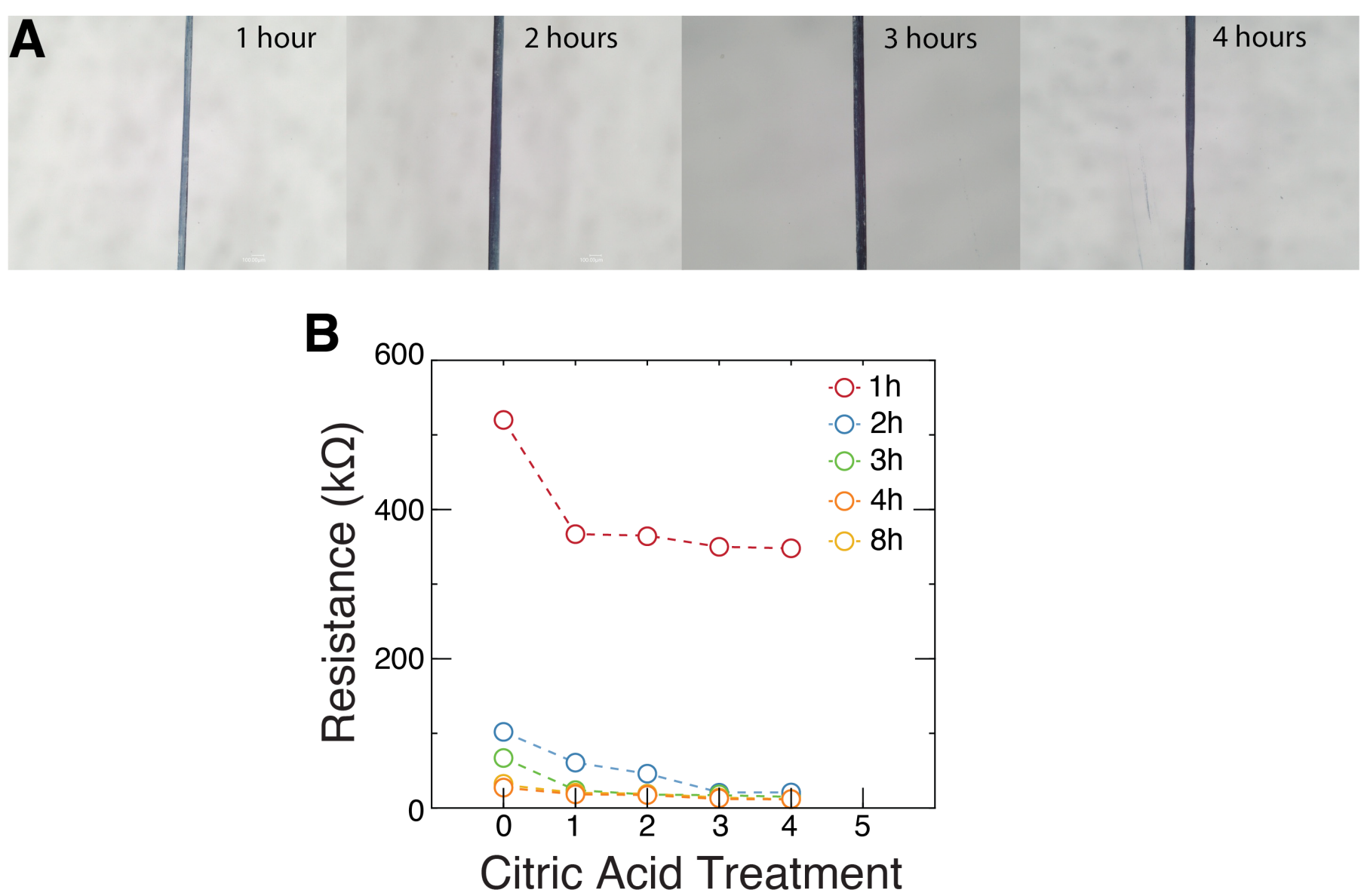

Fig. S15. Coating with D-sorbitol-based gel. (A) Photos of hair color for different coating durations. The initially blond hair darkens over time. **(B)** Resistance versus number of citric acid treatments for hair coated using different polymerization times, as shown in (A). The polymerization occurred for HRP 214 U/ml and EEE-COONa 2 mM and H_2O_2 1 mM. (c = 1 coating cycle).

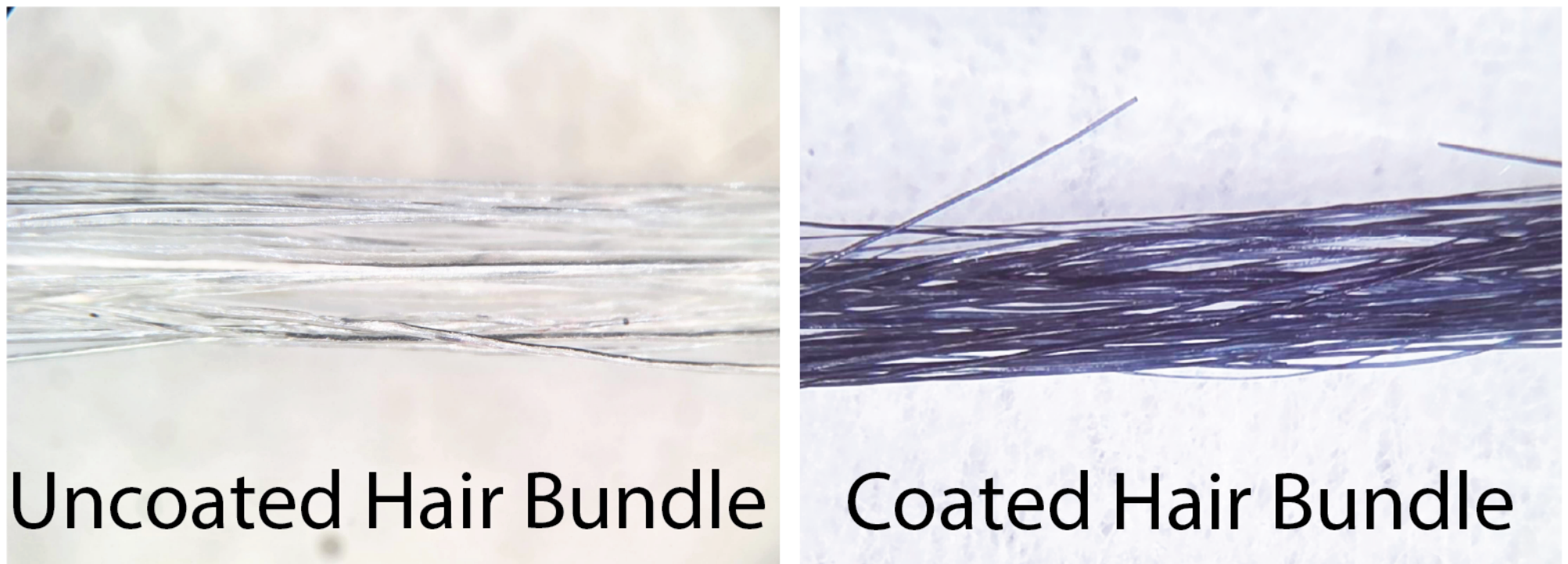

Fig. S16. Hair bundle before and after coating with eDYE$_P$. The coating remains local to each hair shaft even if a bundle is immersed in solution (c = 1 coating cycle).

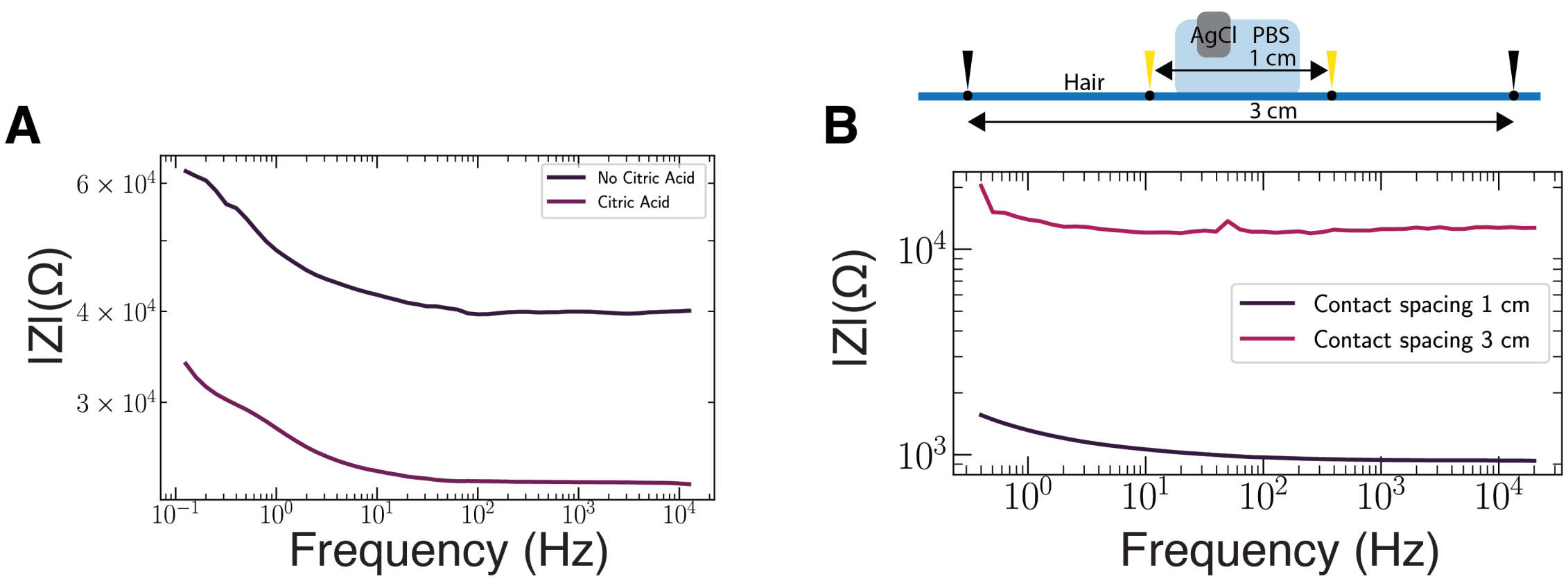

Fig. S17. Parameters that affect electrochemical impedance spectroscopy. (A) EIS for a hair before (black) and after citric acid treatment. Hair length immersed in liquid 4 mm. **(B)** EIS for a hair with different distance between contact points. Hair length immersed in liquid 5 mm. (c = 1 coating cycle).

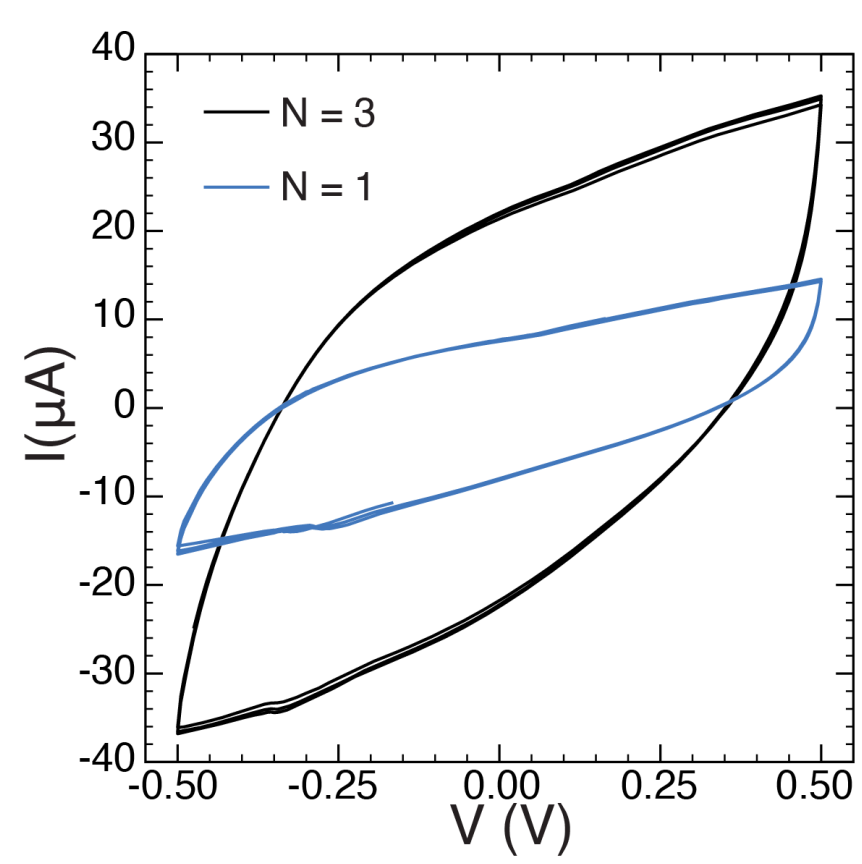

Fig. S18. EDYE$_P$ hair shows capacitive behavior characteristics. Cyclic voltammograms of eDYE$_P$ single-hair (N = 1) and three-hair (N = 3) electrodes, showing capacitive, mixed-conducting behavior characteristic of PEDOT-like OMIECs. (c = 1 coating cycle).

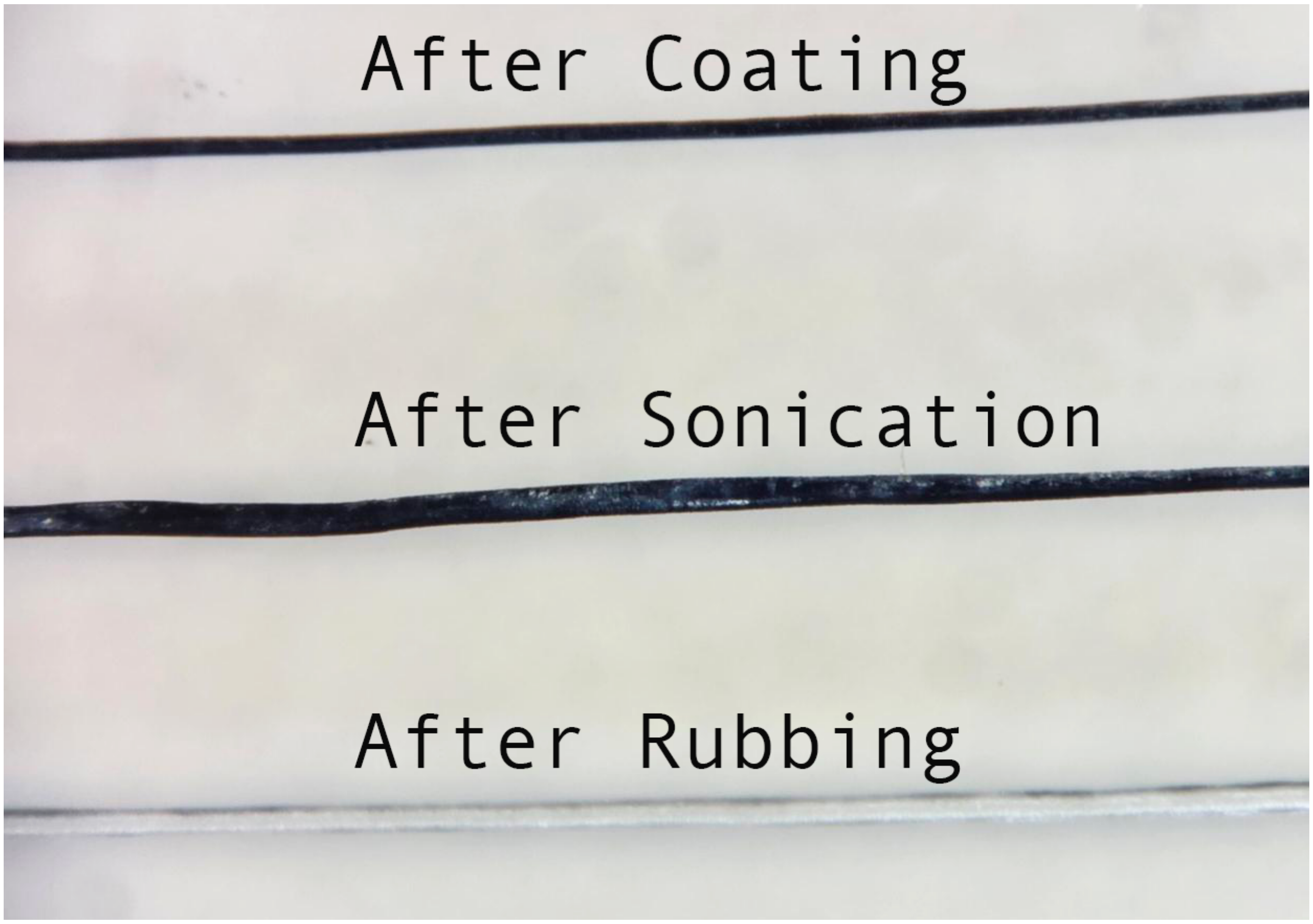

Fig. S19. eDYE$_P$ hair stability under various washing methods. Optical images of eDYE$_P$-coated hair after coating, sonication, and mechanical rubbing.

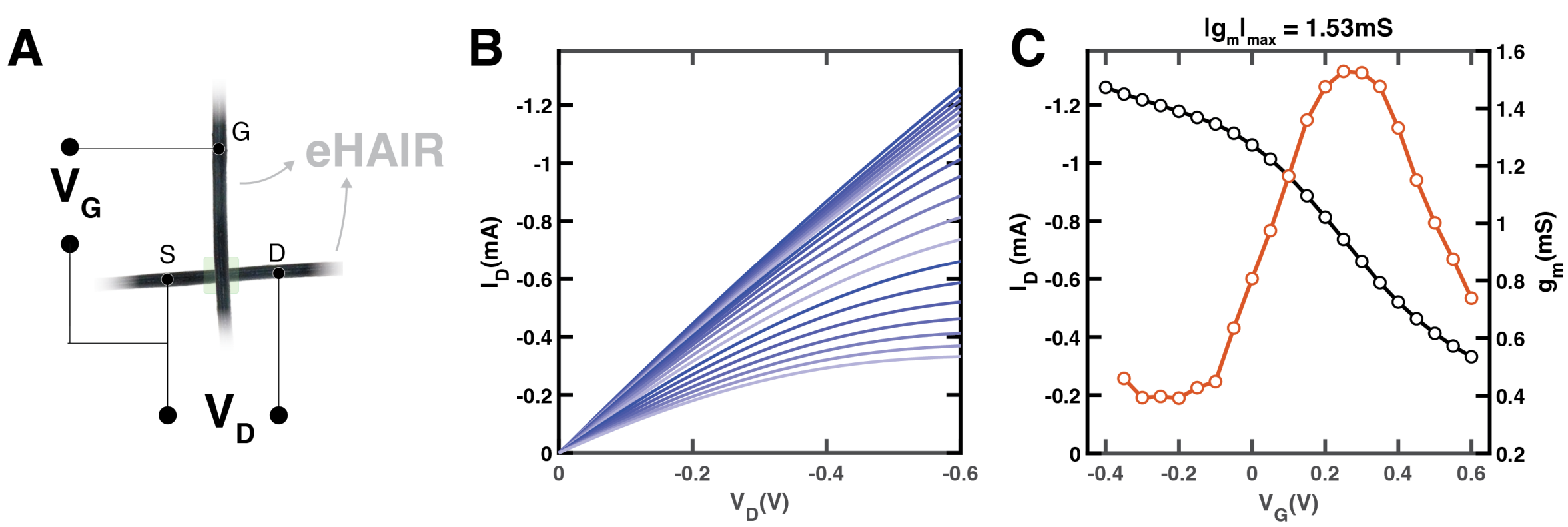

Fig. S20. eHAIR-based OECTs with eHAIR serving as both the channel and the gate. (A) Schematic for e-hair (5 coating cycles) based OECT where an e-hair acts as channel (N=1) and gate (N=1) using PBS as electrolyte. **(B)** Output characteristics (ID – VD) of an e-hair OECT at varying gate voltages from -0.4 V (dark blue) to +0.6 V (light blue) for an e-hair as gate , showing clear linear and saturation regimes characteristic of OECT operation. (L = 1 mm, W = 0.2 mm). **(C)** Transfer and transconductance curves (ID, gm) of the same device (for VD = −0.6 V), demonstrating reversible gate-driven modulation of the channel current and a peak transconductance of 1.53 mS.

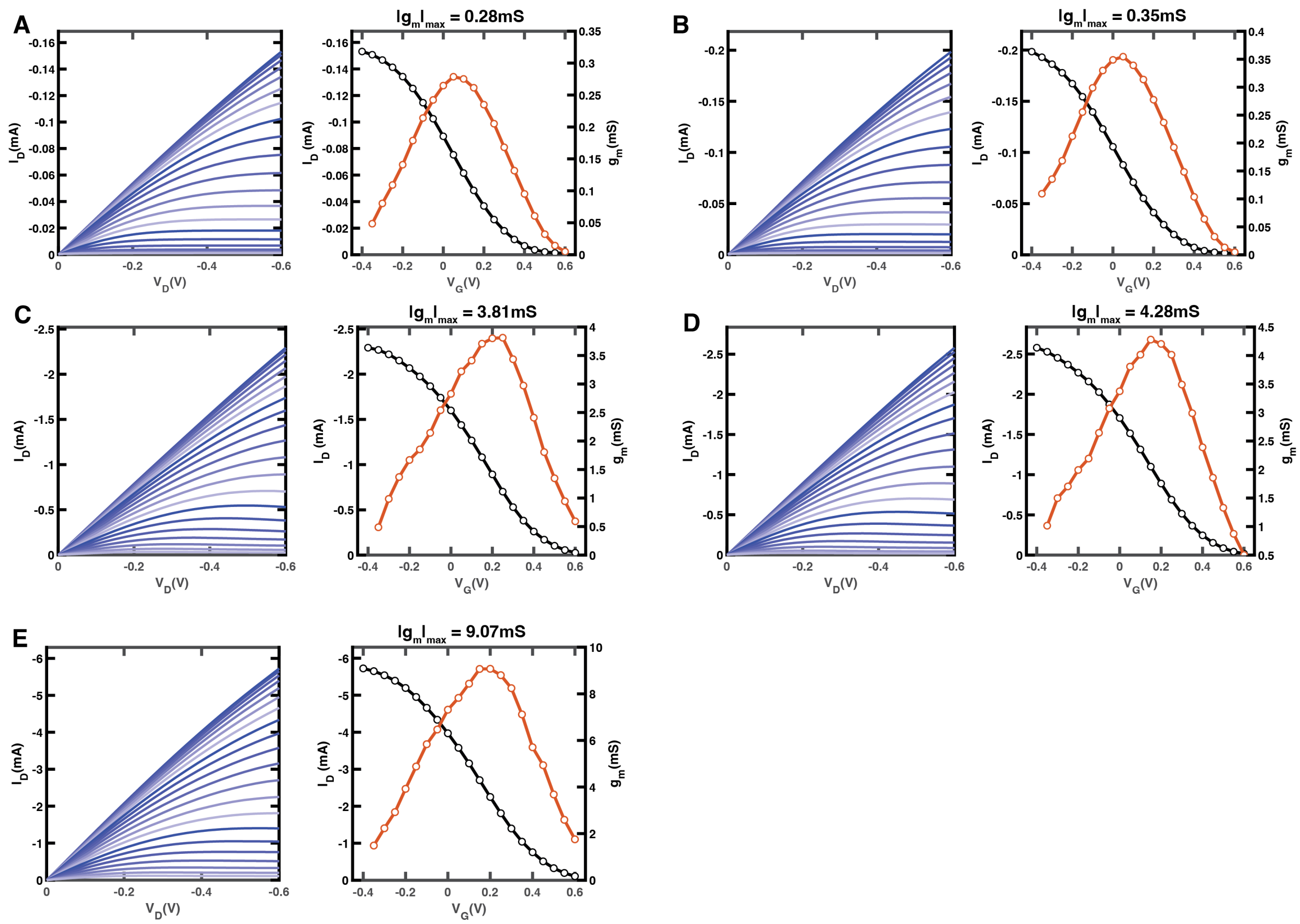

Fig. S21. Transconductance value ($g_{m,max}$) increases with increasing coating thickness. Output characteristics (ID – VD), transfer and transconductance curves (ID, gm) of an eHAIR OECT (L = 1 mm, W = 0.2 mm) for one to five $eDYE_C$ coating cycles (c = 1–5), shown in panels (A) - (E), respectively.

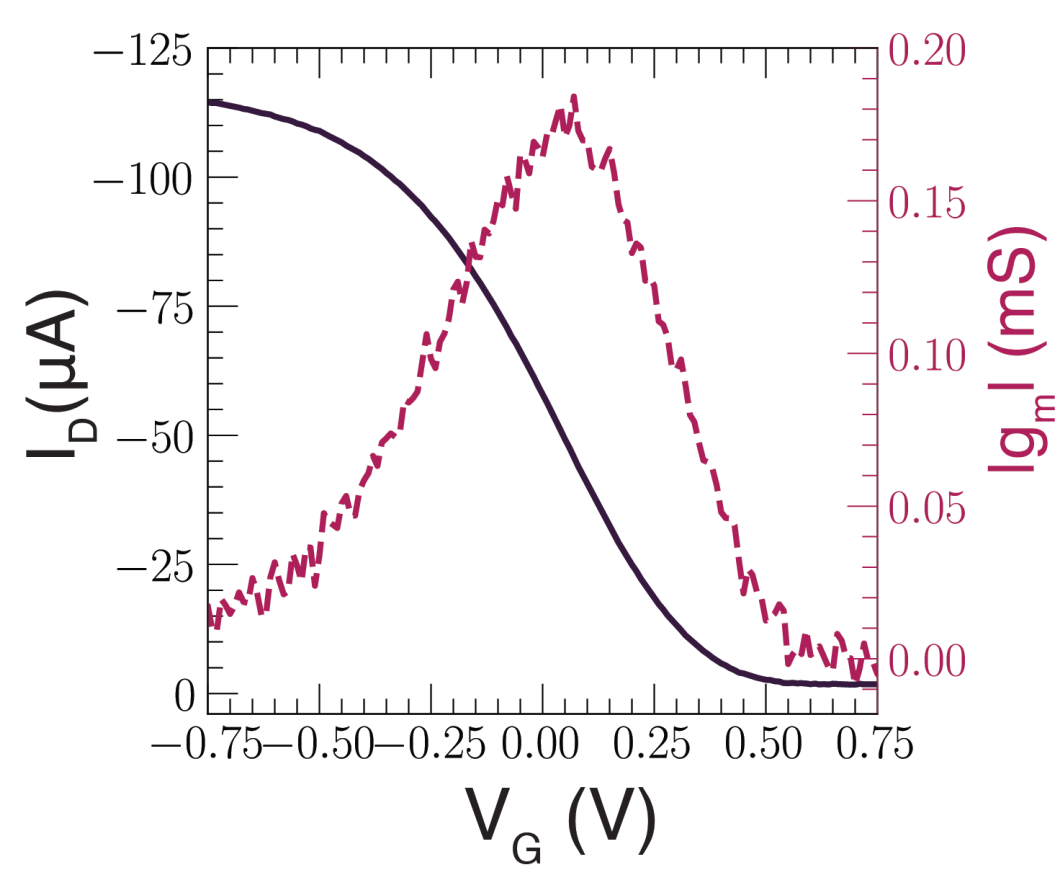

Fig. S22. Transfer curve and corresponding transconductance for a hair OECT based on $eDYE_P$. Transfer curve of an in situ formed coated hair with HRP 5mg/ml (107 U/mg) EEE-

COONa 2 mM and H_2O_2 1 mM. The length of the OECT channel was 5 mm with the channel width equal to the circumference of the hair. (c = 1 coating cycle)

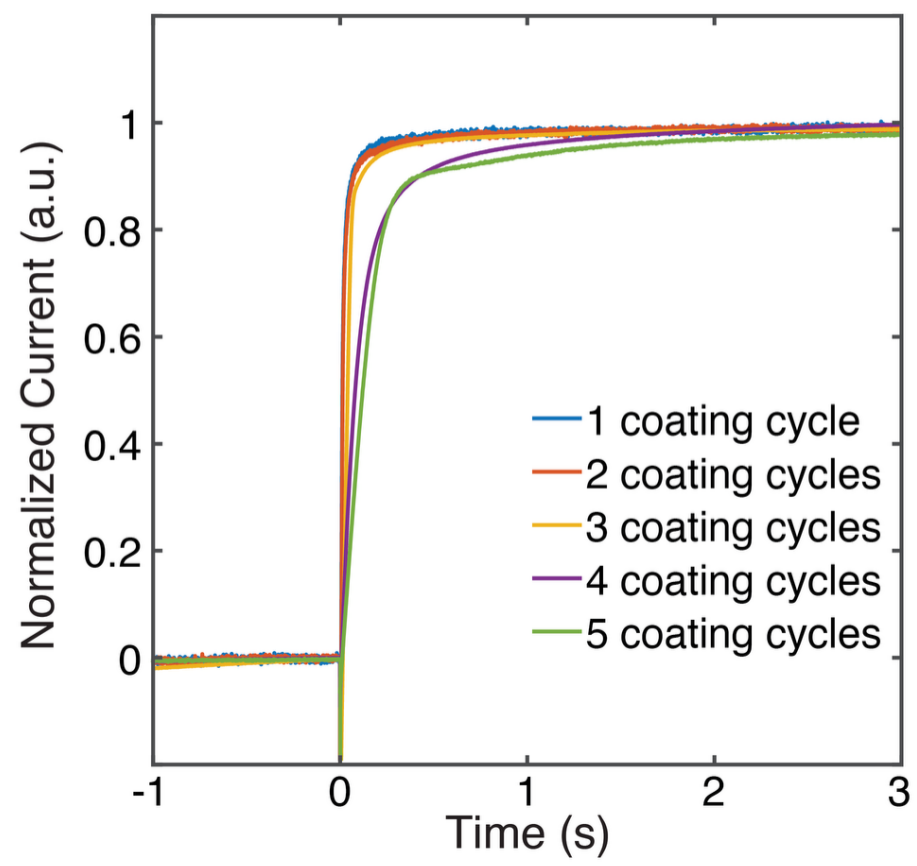

Fig. S23. Effect of coating thickness on the temporal response of eHAIR OECT. Temporal response (τ) of eHAIR OECTs as a function of the number of $eDYE_C$ coating cycles (c = 1–5). Thinner channels exhibit faster responses, while thicker coatings lead to slower ionic dynamics.

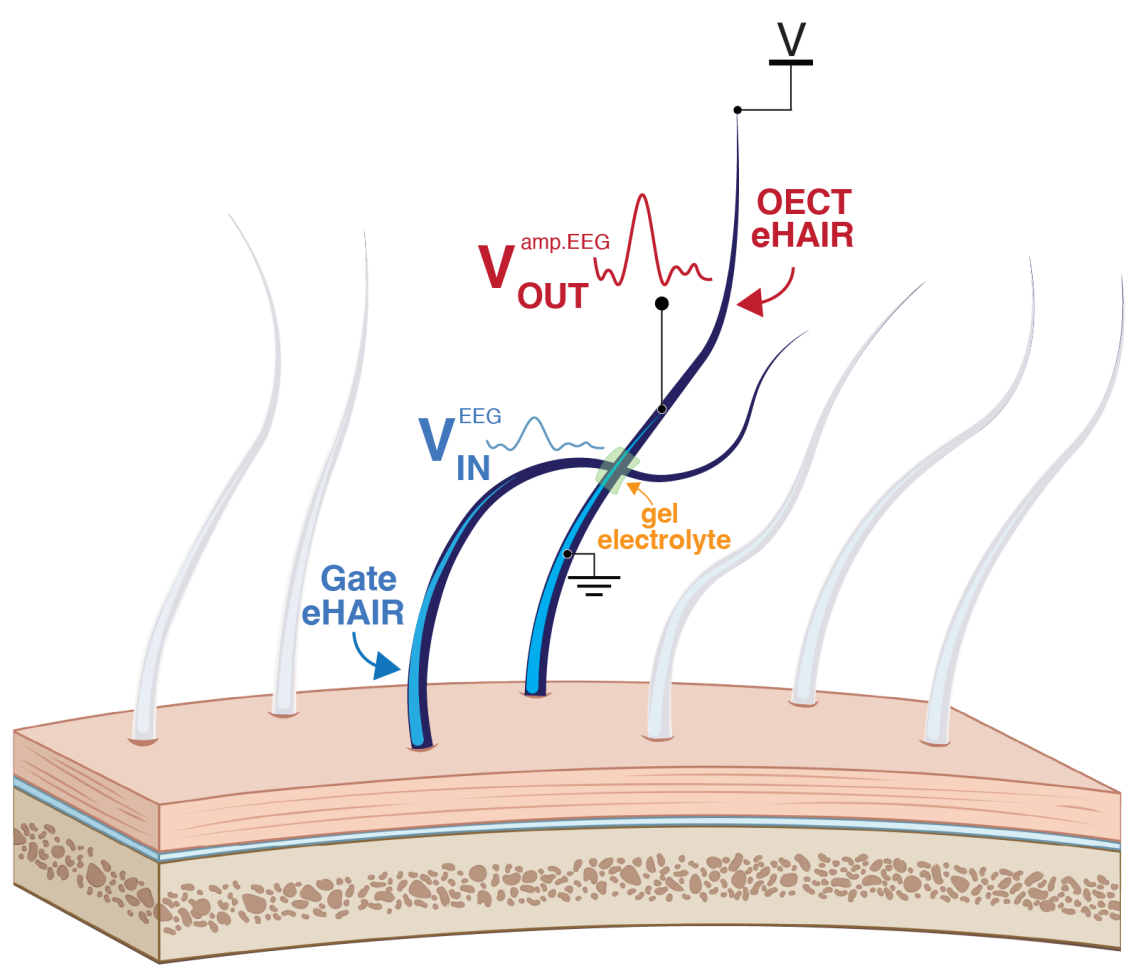

Fig. S24. Conceptual implementation of an eHAIR-based OECT for active EEG recording. A conductive eHAIR strand or hair bundle serves as the gate electrode that senses local EEG potentials and couples them to an adjacent eHAIR OECT through a gel electrolyte. The OECT operates in a common-source amplifier configuration, locally amplifying the electrophysiological signal before transmission to the external acquisition electronics. This schematic illustrates one possible future architecture for hair-integrated active electrophysiological recording systems. Partially created in BioRender. Spyropoulos, G. (2026) https://BioRender.com/jhzhhkx.

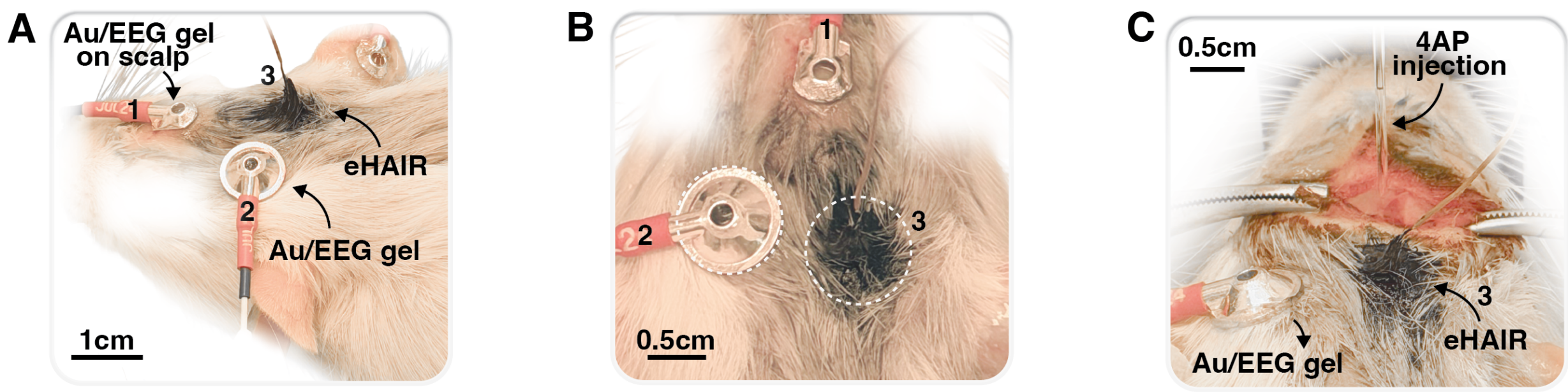

Fig. S25. In vivo electrode configuration for eHAIR and Au/EEG gel EEG recordings. (A) Top-view photograph of the in vivo rat EEG configuration showing simultaneous recordings with an eHAIR (c = 5 coating cycles) electrode and a conventional Au/EEG gel electrode placed on the scalp. **(B)** Close-up view of the electrode arrangement highlighting the spatially confined eHAIR-coated region and the adjacent Au/EEG gel electrode on the scalp. Area: ~64mm^2. **(C)** Surgical view of the rat skull illustrating electrode placement during seizure experiments, with the eHAIR and Au/EEG gel electrodes positioned near the site of intracortical 4-aminopyridine (4-AP) injection.

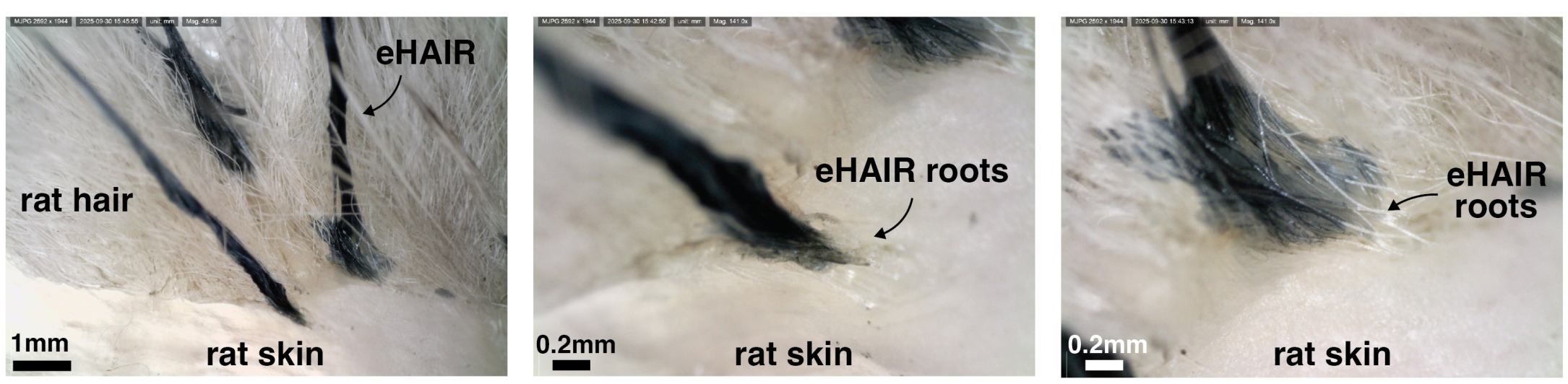

Fig. S26. eDYE$_C$-coated eHAIR-skin interface in rat scalp. Optical micrograph showing eHAIR-coated rat hair (c = 5 coating cycles) interfacing with the skin surface, highlighting the localized conductive coating along the hair shaft and coating extending towards the hair root.

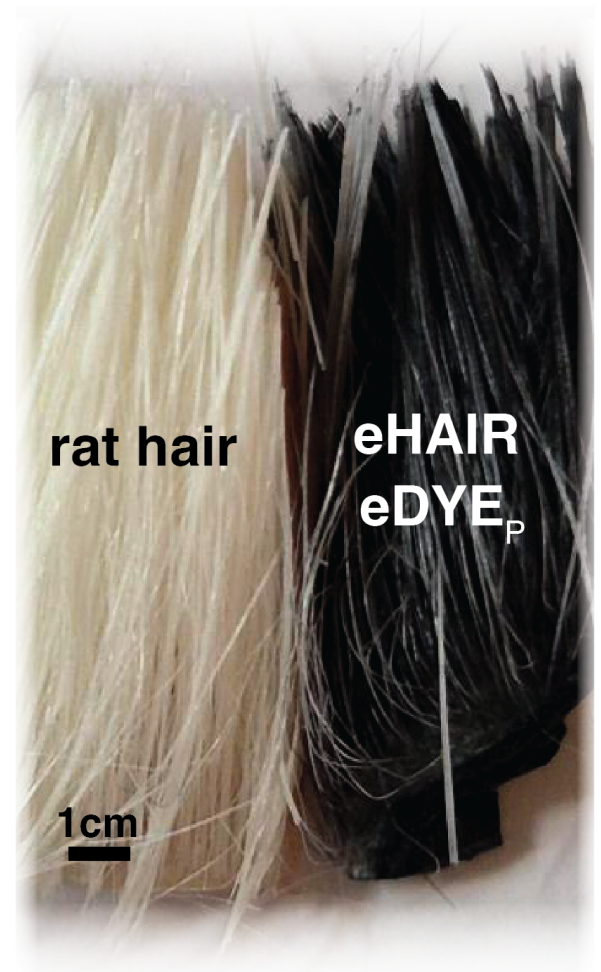

.

Fig. S27. EDYE$_p$-coated rat hair. Optical image comparing native rat hair (left) and eDYE$_P$ coated eHAIR (right), illustrating the uniform darkening and visual contrast induced by the conductive coating (c = 3 coating cycles).

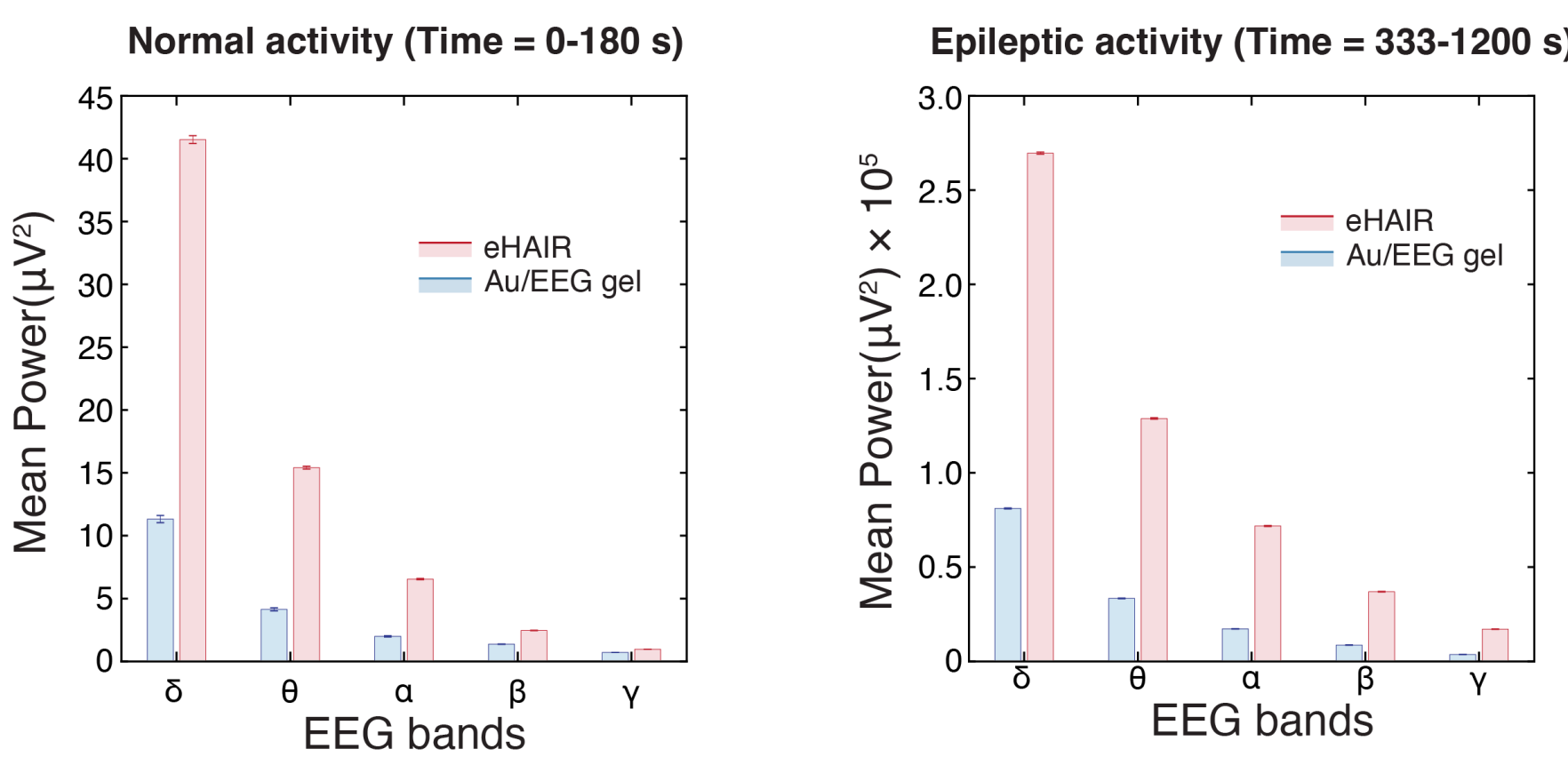

Fig. S28. Band-limited mean power across canonical EEG bands (δ, θ, α, β, γ). eHAIR displays higher mean power in all bands during baseline and seizures.

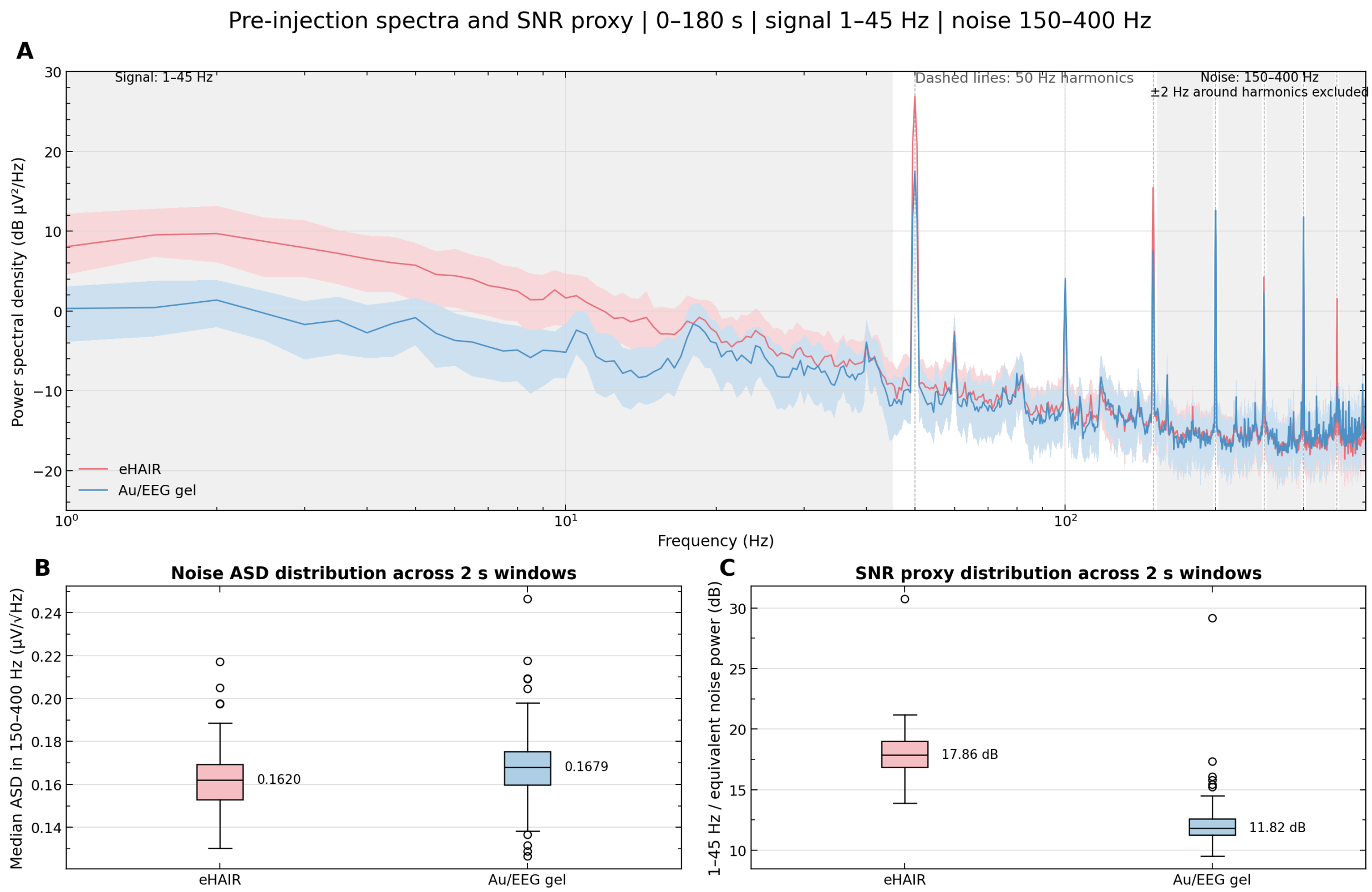

Figure S29. Pre-injection spectral noise and SNR proxy comparison between eHAIR and Au/EEG gel. (A) Median power spectral densities (PSDs) from the 0–180 s pre-injection recording, calculated using 2 s Hann-windowed segments with a 1 s step (179 windows); shaded regions indicate the interquartile range. No notch filter was applied before spectral estimation, and dashed lines mark the 50 Hz mains harmonics. (B) Distributions of noise amplitude spectral density (ASD), calculated for each window as the square root of the median PSD between 150 and 400 Hz after excluding frequency bins within ±2 Hz of the 50 Hz harmonics. (C) SNR-proxy distributions, calculated as $10 \times \log_{10}[P_{1\text{–}45\ \text{Hz}}/(\text{noise PSD} \times 44\ \text{Hz})]$. Boxplots show medians, interquartile ranges, 1.5×IQR whiskers, and outliers. Median noise ASD was 0.1620 µV/√Hz for eHAIR and 0.1679 µV/√Hz for Au/EEG gel; median SNR was 17.86 and 11.82 dB, respectively.

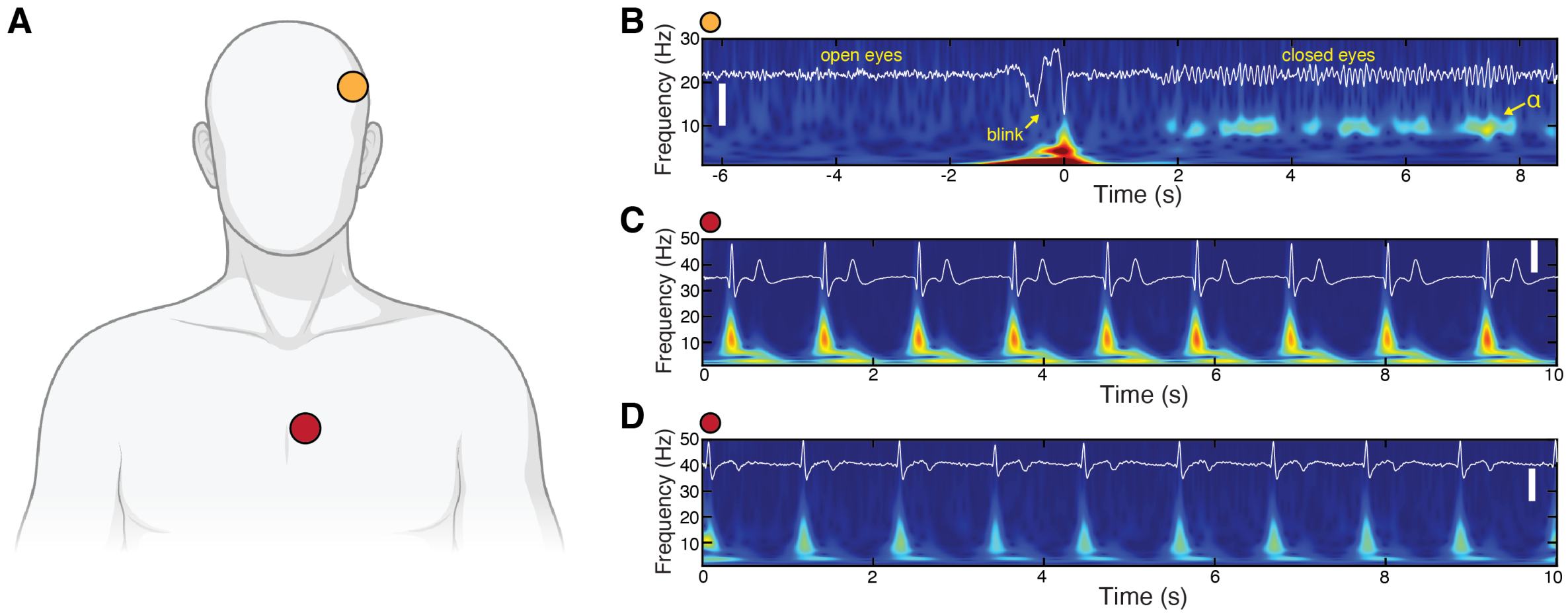

Fig. S30. eHAIR enables high-quality EEG and ECG recording in humans. (A) Placement sites for human EEG (orange marker) and ECG (red marker). **(B)** Human EEG recorded with extracted eHAIR (interface area: 0.8mm^2) coated (c = 5 coating cycles) ex situ with the use of gel electrolyte. Time-domain signal showing strong alpha oscillations during eye closure and a brief blink artifact. Scale bar 200µV. Corresponding spectrogram confirming 8-12 Hz alpha-band dominance and broadband power during ocular events. Scale bar 500µV. **(C)** Human ECG recorded using extracted eHAIR coated (c = 5) ex situ with the use of gel electrolyte. The time-domain signal shows clearly defined P-waves, QRS complexes (~±500 µV), and T-waves. The accompanying spectrogram exhibits periodic bursts of energy aligned with the QRS complexes, confirming stable and high-fidelity cardiac recording. **(D)** Human ECG recorded using extracted e-hair coated (c = 5) ex situ without the use of electrolyte or gel. Fig. S30A was partially created in BioRender. Spyropoulos, G. (2026) https://BioRender.com/t25as7v.

Movie S1. eDYEp coated hair partially immersed in a PBS solution. The hair is acting as a working electrode and an Ag/AgCl electrode immersed in the electrolyte is acting as a reference electrode. Pulsing of the eHAIR shows electrochromic change of color due to doping and de-doping of the conducting polymer coating.

Movie S2. In situ local polymerization of a single hair, pre-functionalized with HRP, immersed in monomer, H_2O_2, and sucrose gel.
